# Comparative Genome Analysis Provides Insights into Both the Lifestyle of *Acidithiobacillus ferrivorans* Strain CF27 and the Chimeric Nature of the Iron-Oxidizing Acidithiobacilli Genomes

**DOI:** 10.3389/fmicb.2017.01009

**Published:** 2017-06-13

**Authors:** Tam T. T. Tran, Sophie Mangenot, Ghislaine Magdelenat, Emilie Payen, Zoé Rouy, Hassiba Belahbib, Barry M. Grail, D. Barrie Johnson, Violaine Bonnefoy, Emmanuel Talla

**Affiliations:** ^1^Aix-Marseille Université, CNRS, LCBMarseille, France; ^2^Laboratoire de Biologie Moléculaire pour l’Etude des Génomes, C.E.A., Institut de Génomique – GenoscopeEvry, France; ^3^CNRS UMR8030, CEA/DSV/IG/Genoscope, Laboratoire d’Analyses Bioinformatiques pour la Génomique et le MétabolismeEvry, France; ^4^College of Natural Sciences, Bangor UniversityBangor, United Kingdom

**Keywords:** *Acidithiobacillus*, iron oxidation, sulfur metabolism, chimeric genome, acidophile, psychro-tolerance

## Abstract

The iron-oxidizing species *Acidithiobacillus ferrivorans* is one of few acidophiles able to oxidize ferrous iron and reduced inorganic sulfur compounds at low temperatures (<10°C). To complete the genome of *At. ferrivorans* strain CF27, new sequences were generated, and an update assembly and functional annotation were undertaken, followed by a comparative analysis with other *Acidithiobacillus* species whose genomes are publically available. The *At. ferrivorans* CF27 genome comprises a 3,409,655 bp chromosome and a 46,453 bp plasmid. *At. ferrivorans* CF27 possesses genes allowing its adaptation to cold, metal(loid)-rich environments, as well as others that enable it to sense environmental changes, allowing *At. ferrivorans* CF27 to escape hostile conditions and to move toward favorable locations. Interestingly, the genome of *At. ferrivorans* CF27 exhibits a large number of genomic islands (mostly containing genes of unknown function), suggesting that a large number of genes has been acquired by horizontal gene transfer over time. Furthermore, several genes specific to *At. ferrivorans* CF27 have been identified that could be responsible for the phenotypic differences of this strain compared to other *Acidithiobacillus* species. Most genes located inside *At. ferrivorans* CF27-specific gene clusters which have been analyzed were expressed by both ferrous iron-grown and sulfur-attached cells, indicating that they are not pseudogenes and may play a role in both situations. Analysis of the taxonomic composition of genomes of the Acidithiobacillia infers that they are chimeric in nature, supporting the premise that they belong to a particular taxonomic class, distinct to other proteobacterial subgroups.

## Introduction

*Acidithiobacillus* species are obligately acidophilic, chemolithoautotrophic Gram-negative bacteria, known for their abilities to extract metals such as copper, uranium, cobalt and gold from mineral ores (biomining) and to remove sulfur compounds (bioremediation) from contaminated industrial effluents, liquid wastes or soils (Jerez, 2009; Johnson, 2014). Numerous *Acidithiobacillus* strains have been isolated from natural and man-made low pH environments in a variety of geo-climatic contexts such as acidic ponds, lakes and rivers, sulfur springs, acid mine/rock drainage waters and mining areas (Nuñez et al., 2016). Based on their 16S rRNA gene sequences, these microorganisms were initially considered to be Gammaproteobacteria (Kelly and Wood, 2000; Garrity et al., 2005) though later, based on comparative multiprotein analysis, they, together with *Thermithiobacillus* spp., were transferred to a new proteobacterial class, the Acidithiobacillia (Williams and Kelly, 2013). Using “gold-standard" criteria and physiological and distinctive morphological traits, the genus *Acidithiobacillus* (*At.*) has been shown to consist of at least seven species, all of which derive energy from the oxidation of elemental sulfur and reduced inorganic sulfur compounds (RISCs) to support their growth. Four species (*At. ferrooxidans, At. ferridurans, At. ferrivorans* and *At. ferriphilus*) also catalyze the dissimilatory oxidation of ferrous iron (Fe(II)), and some species and strains can also use molecular hydrogen as an electron donor (Amouric et al., 2011; Hedrich and Johnson, 2013; Nitschke and Bonnefoy, 2016; Nuñez et al., 2016, 2017).

Iron-oxidizing *Acidithiobacillus* spp. sometimes display distinctive physiological and morphological traits, such as motility, pH- and metal-tolerance, and growth temperatures, but they also differ in the components involved in the Fe(II) and RISCs oxidation pathways (Amouric et al., 2011; Talla et al., 2014; Dopson, 2016; Nitschke and Bonnefoy, 2016). While all the Fe(II)-oxidizing *Acidithiobacillus* spp. examined so far have the *rusA* gene encoding the classical rusticyanin A, only strains of *At. ferrivorans* and *At. ferriphilus* have the iron oxidase-encoding gene (*iro*) (Amouric et al., 2011; Nitschke and Bonnefoy, 2016). In addition, most strains of the latter two species have also the gene encoding an isozyme of rusticyanin (*rusB*). All *At. ferrivorans* strains reported in the literature have been isolated from low-temperature acid mine drainage streams (Blake and Johnson, 2000; Kupka et al., 2007; Hallberg et al., 2010; Barahona et al., 2014), and are capable of oxidizing Fe(II) and RISCs as energy sources in a wide range of growth temperatures (5–30°C, optimal temperature 20–22°C) (Kupka et al., 2007, 2009; Hallberg et al., 2010; Barahona et al., 2014). Catalyzing low-temperature metal sulfide dissolution (Kupka et al., 2007), makes them potentially more suitable for biomining in cold regions. However, *At. ferrivorans* [and also some strains of *At. ferriphilus*; (Falagán and Johnson, 2016)] are psychro-tolerant rather than psychrophilic, and grow optimally at temperatures ∼30°C.

A preliminary study of the *At. ferrivorans* CF27 draft genome, consisting of 82 contigs spanning 3.44 Mbp, was reported by Talla et al. (2014). This strain, and the other (SS3; Liljeqvist et al., 2011) whose genome is currently publically available, are highly related according to 16S RNA gene sequence (99.9% identity) (Amouric et al., 2011). The predicted gene determinants associated to Fe(II) oxidation and RISCs pathways in *At. ferrivorans* CF27 have been identified (Talla et al., 2014). Unlike other *At. ferrivorans* strains studied so far, CF27 does not contain the gene encoding the isozyme RusB, suggesting that this type of rusticyanin is not essential for Fe(II) oxidation. In addition, in contrast to other *Acidithiobacillus* spp. whose genomes are available, *At. ferrivorans* CF27 has a cluster of genes involved in fucose biosynthesis, which could explain why this strain has been frequently observed to form macroscopic biofilms that coagulate mineral particles in liquid media (Talla et al., 2014).

In this work, we report a refined genome sequence assembly of *At. ferrivorans* CF27 as well as its structural and functional annotation. A comparative study of their genomes revealed that *At. ferrivorans* strains CF27 and SS3 harbor a high proportion of strain-specific genes (mainly ‘hypothetical’ or ‘protein of unknown function’), indicating a potential high variability of gene content in *At. ferrivorans* genomes. To gain insight into these ‘hypothetical’/‘protein of unknown function’-encoding genes, clusters of genes specific to *At. ferrivorans* CF27 were identified and the expression of at least one gene of each cluster was analysed in Fe(II)-grown cells and sulfur-attached cells. Finally, phylogenomics analysis highlighted the chimeric taxonomic composition of the genomes of the members of the Acidithiobacillia class, and thus confirmed that this taxonomic group belongs to a particular taxonomic class, distinct from other Proteobacteria.

## Materials and Methods

### Strain and Growth Conditions

*Acidithiobacillus ferrivorans* strain CF27, originally isolated from an acidic stream draining an abandoned Co/Cu mine in Idaho, United States (Blake and Johnson, 2000; Hallberg et al., 2010), was grown routinely in a liquid medium containing basal salts and trace elements (Nancucheo et al., 2016) and either 20 mM ferrous iron (at an initial pH of 1.9), or 1% (w/v) elemental sulfur (initial pH 3.0), incubated, with shaking, at 30°C (Hallberg et al., 2010).

### Genomic DNA Preparation and PCR

DNA from *At. ferrivorans* CF27 was extracted from 5 ml of Fe(II)-grown cells as (described in Osorio et al., 2013). PCR amplifications were carried out with GoTaq G2 Flexi DNA polymerase (Promega) using genomic DNA from *At. ferrivorans* CF27 as template, following the manufacturer’s instructions. The PCR program was as follows: initial denaturation at 94°C for 2 min 30 s, 30 cycles of (i) denaturation for 30 s at 94°C, (ii) annealing for 30 s at 58°C or 60°C, depending on the primers, and (iii) elongation at 72°C for 30 s or 1 min, depending on the size of the amplicon, and a final elongation step of 2 min 30 s at 72°C before the temperature was reduced to 4°C. When necessary, in the RT-PCR experiments, cDNA of the expected size was picked from the corresponding band on an agarose gel, PCR amplified with the same oligonucleotides used for the RT-PCR experiments, before being concentrated and purified using Amicon^®^ Ultra-0.5 centrifugal filter units (Millipore). Nucleotide sequence of the amplified DNA was determined by GATC Biotech (Germany).

### RNA Manipulations

Total RNA from planktonic cells was prepared by using acid-phenol extraction (Aiba et al., 1981) modified according to (Osorio et al., 2013). An additional DNAse I treatment was performed with the reagents from a Turbo DNA-free kit (Applied Biosystems). Total RNA from sulfur-attached cells was prepared according to (Mamani et al., 2016). The RNA integrity was assessed by agarose gel electrophoresis, its purity monitored by measuring optical density (230, 260, and 280 nm) and absence of DNA contamination was checked by PCR on each RNA sample.

Coupled RT-PCR experiments were performed using total RNA extracted from Fe(II)-grown cells and sulfur-attached cells with the Access RT-PCR System (Promega) according to the manufacturer’s instructions. The primers used are described in Supplementary Table S1. For each RT-PCR experiment, the hybridisation (55–66°C) and elongation (68 or 72°C) temperatures, RNA concentration (from 0.1 to 20 ng), DNA polymerase (GoTaq or Tfl from Promega), and the number of cycles (30, 35, or 40) were adjusted. Three controls were used: one without template to detect potential contaminations, one with genomic DNA as a positive control for PCR amplification and one with RNA not treated with reverse transcriptase to check for DNA contamination during RNA preparation.

### DNA Sequencing, Assembling and Genome Analysis

A draft genome of *At. ferrivorans* CF27, consisting of 82 contigs (N_50_ contig size = 218,640 bp), has been previously described (Talla et al., 2014). This preliminary version was based on three genomic libraries, i.e., two pair-end libraries [obtained after DNA shearing to generate 300–600 bp fragments using NEB protocols (New England Biolabs^1^) and SPRIworks HT reagent kit (Beckman Coulter^2^), respectively], and one mate-paired library of ∼5 Kbp fragments using Illumina mate pair library kit (Illumina, San Diego, CA, United States). From these libraries, 100 and 250 bp sequence reads were obtained from HiSeq2000 and HiSeq2500 sequencers (Illumina, San Diego, CA, United States), respectively. In order to refine the assembly, a fourth genomic library, made of ∼10 Kbp fragments, was generated (using the 2D SQK-MAP0005 kit, Oxford Nanopore Technologies^3^) and sequenced with an R7.3 MinION flow cell (FLO-MAP003), and yielded sequence reads of ∼7,810 bp in average. Whole-genome assembly of the *At. ferrivorans* CF27 genome was performed using Newbler assembler v2.8 (Roche, Branford, CT, United States) for Illumina reads, and with SPAdes genome assembler tool^4^ for both Illumina and Nanopore reads. Five of the six obtained contigs were organized into one scaffold (the chromosome) through the comparison between Newbler and SPAdes assemblings. The last contig has the structural and genetic properties of a plasmid amplicon. Therefore, the updated *At. ferrivorans* CF27 genome consists of 6 contigs (N_50_ contig size of 934,903 bp). Note that ∼0.5% nucleotide difference was observed between the draft (with 82 contigs) and update (this work) genomes. All general aspects of the library construction, sequencing and assembly were performed at the Genoscope^5^ (Evry, France). Computational prediction of coding sequences (CDS) and other genome features (RNA encoding genes, ribosome binding sites, etc.), together with functional assignments were performed using the annotation pipeline implemented in the MicroScope platform^6^ (Vallenet et al., 2017). The genes discussed in the text were checked and manually annotated. Accession numbers from the European Nucleotide Archive (ENA^7^) of the updated *At. ferrivorans* CF27 genome sequence are LT841305 for chromosome and LT841306 for the plasmid.

Genomic islands (GIs) in the *At. ferrivorans* CF27 genome were detected using the web server IslandViewer3^8^ (Dhillon et al., 2015) using the GI prediction methods IslandPath-DIMOB (Hsiao et al., 2005), SIGI-HMM (Waack et al., 2006), and IslandPick (Langille et al., 2008), with default parameters. A DNA region was considered as a GI if predicted by at least one of the three prediction methods. GC content and GC skew were calculated using *infoseq* (from EMBOSS package^9^) and in-house perl scripts (Supplementary Text), respectively. Circos software version 0.69 (Krzywinski et al., 2009) were used to produce circular maps representations of the chromosome and plasmid.

### Genome Datasets

Three distinct genome datasets were used in this study. Dataset 1 was composed of seven annotated genomes of *Acidithiobacillus* spp. [*At. ferrivorans* CF27 (this study), *At. ferrivorans* SS3 (Liljeqvist et al., 2011), *At. ferrooxidans* ATCC 23270^T^ (Valdes et al., 2008), *At. ferrooxidans* ATCC 53993 (NC_011206), *At. thiooxidans* ATCC 19377^T^ (Valdes et al., 2011), and *At. caldus* strain SM-1 (You et al., 2011) (all downloaded from the MicroScope website), and *At. caldus* ATCC 51756^T^ (Valdes et al., 2009) retrieved from the NCBI database]. Dataset 2, downloaded from the NCBI ftp site^10^ (In March 2015), consisted of 2,770 complete prokaryotic genomes, excluding those of *Acidithiobacillus* spp. but including 2,605 bacterial and 165 archaeal genomes as well as their taxonomy lineages. Among the bacterial genomes, twenty representatives were chosen from Alpha-, Beta-, Gamma-, or Delta-proteobacteria (Supplementary Table S2) taxonomic classes, to reflect the Proteobacteria phylum. Dataset 3 was the non-redundant prokaryotic database (*nr* prokaryotic database), downloaded from the NCBI ftp site.

### Comparative Genomic Analysis

Taxonomic affiliations from top-scoring BLAST hits were performed as follows. Ribosomal protein sequences were first extracted from *Acidithiobacillus* spp. and from representative proteobacterial proteomes using the *extractseq* (*EMBOSS* package). Next, each protein was searched against the complete prokaryotic dataset 2 proteins (without *Acidithiobacillus* proteins) using the Blastp program (Zhaxybayeva et al., 2009). The highest-ranking match of each protein consists of the best blast hit from an organism that is different from the studied species and having a BLASTP alignment *E*-value less than 10^-5^. The taxonomic assignment of the highest-ranking match was done using the NCBI taxonomy database (Federhen, 2012). The same procedure was applied to each protein of *Acidithiobacillus* spp. and representative proteobacterial proteomes.

Protein classification into gene families included the seven *Acidithiobacillus* spp. (see above). Protein-CDS from *Acidithiobacillus* spp. dataset were clustered in orthologous groups (OG) using OrthoMCL 1.4 (Li et al., 2003) with *E*-value less than 10^-5^, and a default MCL inflation parameter of 1.5. Following this, core OG families (i.e., conserved proteins in all of the seven strains), dispensable OG families (i.e., common proteins present in two or more, but less than seven genomes) and specific OG proteins families (i.e., specific protein groups found in only one genome) were defined. In order to identify cluster regions of specific genes, *At. ferrivorans* CF27-specific OG proteins were searched against the *nr* prokaryotic database using the Blastp program with a threshold *E*-value of 10^-5^, leading to a final set of *At. ferrivorans* CF27-specific genes. We focused our analysis on cluster containing at least three adjacent specific genes.

## Results and Discussion

### Genome Features of *At. ferrivorans* CF27

**FIGURE 1:**
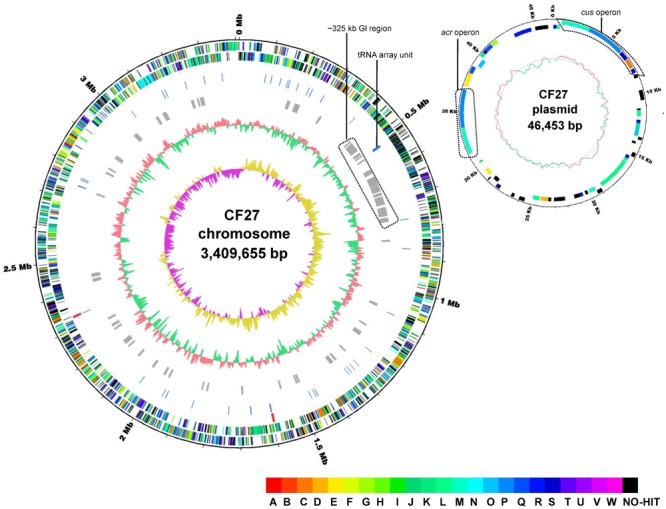
Circular representation of the *At. ferrivorans* CF27 chromosome (A) and plasmid (B). The closed loop chromosome **(A)** provides the following information (from outside to inside): (1) position (in megabases); (2) forward strand CDSs; (3) reverse strand CDSs (colors indicating the assigned COG classes); (4) rRNAs (red); (5) tRNAs (blue); (6) predicted genomic islands; (7) G + C content (red indicates higher G + C compared with the chromosome average G + C content and green indicates lower G + C content); (8) GC skew (purple indicates positive values and yellow, negative values). The tRNA array unit as well as the ∼325 Kbp genomic island (GI) region are shown. The closed loop plasmid **(B)** shows (from outside to inside): (1) position (in megabases); (2) forward strand CDSs; (3) reverse strand CDSs (colors indicating the assigned COG classes); (4), G + C content (red indicates higher G + C compared with the plasmid average G + C content and green indicates lower G + C content). Multi-drug efflux system (*acr*) and copper resistance (*cus*) operons are shown. COG categories are shown in different colors (see the COG color legend) and are associated with the corresponding capital letters: A, RNA processing and modification; B, chromatin structure and dynamics; C, energy production and conversion; D, cell cycle control, cell division, and chromosome partitioning; E, amino acid transport and metabolism; F, nucleotide transport and metabolism; G, carbohydrate transport and metabolism; H, coenzyme transport and metabolism; I, lipid transport and metabolism; J, translation, ribosomal structure, and biogenesis; K, transcription; L, replication, recombination, and repair; M, cell wall/membrane/envelope biogenesis; N, cell motility; O, posttranslational modification; protein turnover, chaperones; P, inorganic ion transport and metabolism; Q, secondary metabolites biosynthesis, transport, and catabolism; R, general function prediction only; S, function unknown; T, signal transduction mechanisms; U, intracellular trafficking, secretion, and vesicular transport; V, defense mechanisms; W, extracellular structures; NO-HIT, proteins not belonging to COG categories.

Preliminary analysis of the draft genome of *At. ferrivorans* CF27, consisting of 82 contigs with a low N_50_ contig size of 218,640 bp, had identified genes coding for Fe(II)- and RISCs-oxidation pathways, and biofilm formation (Talla et al., 2014). By using Nanopore technology for sequencing and new assembly strategy, the *At. ferrivorans* CF27 genome, contained on the chromosome of 3,409,655 bp and one plasmid of 46,453 bp, was refined, yielding a new and updated version (6 contigs with N_50_ contig size of 934,903 bp) (**Figure 1**). The general features of the updated *At. ferrivorans* CF27 genome were compared with that of other *Acidithiobacillus* spp. whose genomes are available (Supplementary Table S3). As previously reported (Talla et al., 2014), the current version of the *At. ferrivorans* CF27 genome also harbors a GC content of 56.5%. With the updated *At. ferrivorans* CF27 genome, an additional ribosomal RNA (*rrn*) operon was found, leading to two rRNA operons organized in the order 16S-23S-5S, as in other *Acidithiobacillus* spp. (**Figure 1**, circle 4; Supplementary Table S3). Similar to *At. ferrooxidans* ATCC 23270^T^, *At. ferrivorans* CF27 has a high number (73 in its chromosome) of tRNA genes, of which 26 are clustered in a specific region (tRNA array unit) (**Figure 1**, circle 5), which has been acquired through horizontal gene transfer (HGT), possibly from acidophilic Firmicutes, before being subjected to tRNA rearrangements, deletions, insertions, and duplications (Tran et al., 2015). Fifty-one GIs ranging from 4.2 to 33.5 Kbp (490.4 Kbp in total; 14.4% of the total genome) were identified in the *At. ferrivorans* CF27 genome (Supplementary Table S4). Most display a GC content between 42.8 and 64.1% (average of 54.4%) and encode putative proteins involved in several functions (type II and IV secretion systems, putative N6-adeinine specific methyltransferase, CRISPR-associated protein Cas2, etc.) as well as unknown or hypothetical proteins (**Figure 1**, circle 6; Supplementary Table S4). Interestingly, the gene cluster proposed to be involved in the formation of macroscopic biofilms in *At. ferrivorans* CF27 (Talla et al., 2014) is located in the GI51, suggesting that these genes have also been acquired by HGT. In addition, the analysis of the updated *At. ferrivorans* CF27 genome sequence has shown that the tRNA array unit is located within a ∼325 Kbp genomic segment containing numerous GIs (GI4-GI16) (**Figure 1**, circle 6; Supplementary Table S4). This observation provides additional evidence for acquisition of this tRNA array unit by lateral gene transfer, in agreement with what has been observed in *At. ferrooxidans* ATCC 23270^T^ in which the tRNA array unit was also located in a putative integrative conjugative element (ICE) of 300 Kbp (Levican et al., 2009).

The updated *At. ferrivorans* CF27 genome exhibits 3,888 predicted coding sequences (CDS) from which 2,515 (64.7% of the total) could be classified in at least one of the Clusters of Orthologous Groups (COG). The distribution of the COG functional classes in the chromosome and the plasmid is given in Supplementary Table S3. The five most abundant functional categories within *Acidithiobacillus* spp. were ‘Replication Recombination and Repair (L),’ ‘Cell wall/membrane/envelope biogenesis (M),’ ‘Amino acid transport and metabolism (E),’ ‘Energy production and conversion (C)’ and ‘Inorganic ion transport and metabolism (P),’ accounting for ∼30% of the overall CDS (Supplementary Table S3). These observations could reflect the specific adaptation of *Acidithiobacillus* spp. to mining environments that contain elevated concentrations of soluble transition metals but relatively low amounts of dissolved organic carbon.

The plasmid (**Figure 1**) encodes 50 putative CDS, including genes predicted to be involved in plasmid replication (i.e., *repA*, AFERRI_v1_p0010) and mobilization (*mob*, AFERRI_v1_p0021), in toxin–antitoxin systems [p0001-p0002, p0019-p0020 and p0025 (toxin)] as well as in copper resistance (*cus* operon, p0003-p0005) and a multi-drug efflux system (*acr* operon, p0039-p0041). Interestingly, the gene synteny of the operons involved in copper and multidrug efflux systems (see downstream) coded by the *At. ferrooxidans* CF27 plasmid is similar to that observed in the plasmids identified in *At. caldus* SM-1, suggesting a similar origin.

### *At. ferrivorans* CF27 Has Genes Allowing Its Adaptation to Harsh Environments

#### Cold-Adapted Lifestyle

Most of the genes proposed to be involved in cold adaptation in *At. ferrivorans* strain SS3 were also detected in strain CF27 (Liljeqvist et al., 2015; Christel et al., 2016) (Supplementary Table S5). These include the genes for (i) compatible solute synthesis and assimilation, such as sucrose and trehalose (**Supplementary Figure S1**); (ii) cold shock proteins (CSP) to maintain cell integrity and metabolism, in particular nucleic acid binding chaperones; (iii) stabilization of transcripts; (iv) folding of newly synthesized proteins; (v) membrane fluidity maintenance.

#### Metal(loid) Resistance

The ability of *At. ferrivorans* strains to tolerate elevated concentrations of transition metals appears to be significantly lower than that of other iron-oxidizing acidithiobacilli (Hallberg et al., 2010; Hedrich and Johnson, 2013; Falagán and Johnson, 2016), though it is much greater than that reported for most other prokaryotes. The genetic determinants involved in metal resistance in *At. ferrooxidans* CF27 were detected in its genome.

We identified 17 genes predicted to be involved in copper efflux (**Supplementary Figure S2** and Table S5). These genes encode (i) a putative cytoplasmic copper chaperone CopZ and two copper-exporting P-type ATPases (CopAB) that pump copper from the cytoplasm to the periplasm; (ii) three RND-copper efflux systems (CusCBA) bridging inner- and outer membranes allowing extrusion of copper and silver from the cytoplasm or the periplasm to the extracellular space; (iii) a putative phosphate transporter (Pho84) able to extrude Cu-phosphate complexes formed after polyphosphate hydrolysis by exopolyphosphatase.

*Acidithiobacillus ferrivorans* CF27 also encodes Zn^2+^ export systems (**Supplementary Figure S2** and Table S5): (i) four cation diffusion facilitators (CDF) transporters (CzcD) and (ii) possibly at least one of the Cus efflux system described above, since the CusCBA and the RND-Cd^2+^, -Zn^2+^ and -Co^2+^ efflux system (CzcCBA) are highly similar and could be mixed up.

Manganese ions (Mn^2+^) are likely removed from the cell through four predicted CorA transport systems (**Supplementary Figure S2** and Table S5), also known to be involved in the regulation of Co^2+^ efflux. The *cor* cluster remains conserved in terms of synteny, gene content and organization within *Acidithiobacillus* spp., except in strains of *At. caldus* (**Supplementary Figure S2**) for which the cluster is composed of five genes.

Concerning mercury resistance, *At. ferrivorans* CF27 genome has most of the *mer* genes involved in regulation, mercuric reduction, binding and transport (**Supplementary Figure S2** and Table S5). However, we did not find genes predicted to encode the additional regulatory protein (*merD*) and the mercuric ion transporter (*merC*) present in the *mer* operon in other *At. ferrooxidans* strains (Valdes et al., 2008), nor the gene encoding the organomercury lyase (*merB*) located within the *mer* operon of *At. caldus* SM-1 (You et al., 2011). Together, these analyses highlight the diversity of structure and organization of *mer* operons in the acidithiobacilli.

*Acidithiobacillus* spp. are able to tolerate arsenic through an efflux system coding by arsenic resistance (*ars*) genes. While the *arsBRC* operon was found in all *At. caldus* and in *At. ferrooxidans* ATCC 23270^T^ (Valdes et al., 2008; Dopson and Holmes, 2014), an *arsRCDA* operon was identified in *At. ferrivorans* CF27 genome that share synteny with that described in *At. ferrivorans* SS3 genome (**Supplementary Figure S2**). In addition, genes encoding the arsenite transporter ArsB and another arsenate reductase ArsC, were also detected elsewhere in the *At. ferrivorans* CF27 genome (Supplementary Table S5). Taken together, these results show that *At. ferrivorans* CF27 exhibits all the genetic determinants required to make it tolerant to heavy metal(loid)s, including copper, silver, zinc, cadmium, cobalt, manganese, mercury and arsenic.

#### Motility and Chemotaxis

*Acidithiobacillus ferrivorans* CF27 has been described as highly motile (Hallberg et al., 2010) and accordingly its genome contains a large cluster (**Supplementary Figure S3**) with at least 32 genes involved in flagella biosynthesis and assembly (Supplementary Table S5). A model of the flagellar system of *At. ferrivorans* CF27 is illustrated in **Supplementary Figure S3**. To control the direction of the rotation of the flagella, *At. ferrivorans* CF27 contains a gene cluster involved in chemotaxis (**Supplementary Figure S3** and Table S5). This cluster was found downstream of the genes involved in the flagella biosynthesis and assembly. Interestingly, this locus was partially located within a genomic island (GI48) and was not detected in *At. ferrivorans* SS3 suggesting that the latter strain is not motile, contrarily to the other strains of this species described so far (Hallberg et al., 2010; Barahona et al., 2014). Moreover, each of flagella and chemotaxis gene clusters (**Supplementary Figure S3**) exhibit gene synteny patterns with *At. thiooxidans* and two strains of *At. caldus* (SM-1, ATCC 51756^T^), which suggests that they were acquired through HGT in *At. ferrivorans* CF27, *At. caldus* and *At. thiooxidans* from the same ancestor or could be present within the *Acidithiobacillus* common ancestor and lost in *At. ferrivorans* SS3 and *At. ferrooxidans* strains.

### *At. ferrivorans* Strains CF27 and SS3 Harbor High Proportions of Strain-Specific Genes

**FIGURE 2:**
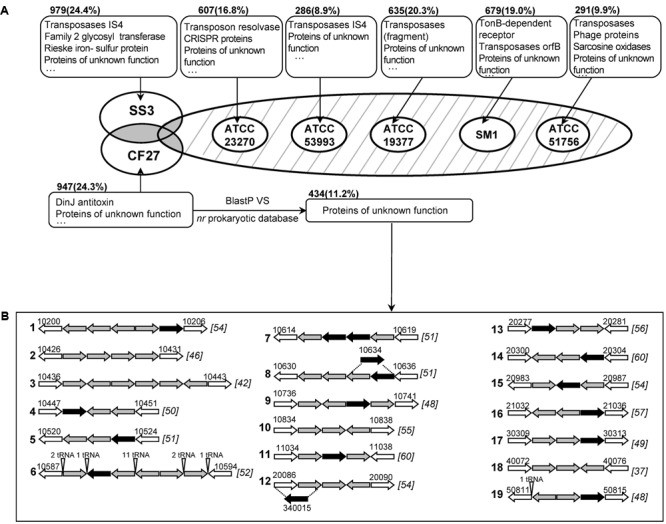
**(A)**Venn diagram of orthologous groups (OG) from seven *Acidithiobacillus* genomes. Only strain-specific genes are displayed. The strain names are: CF27, *At. ferrivorans* CF27; SS3, *At. ferrivorans* SS3; ATCC 23270, *At. ferrooxidans* ATCC 23270^T^; ATCC 53993, *At. ferrooxidans* ATCC 53993; ATCC 19377, *At. thiooxidans* ATCC 19377^T^; SM1, *At. caldus* SM-1; and ATCC 51756, *At. caldus* ATCC 51756^T^. The main functional proteins are shown in boxes. The number of strain-specific genes is shown with the percentage (compared to the corresponding proteome) in the parentheses. Common genes between *Acidithiobacillus* species (shaded in gray or with hatching lines) are described in **Supplementary Figure S4**. **(B)** Gene organization of specific clusters in *At. ferrivorans* CF27. Specific gene clusters are limited by non-specific genes (in white arrows) with their locus names indicated above. Successful specific gene expression obtained by RT-PCR is shown as black arrow. Clusters are numbered according to their location along the *At. ferrivorans* CF27 genome. The G + C content (%) of the overall gene cluster is shown in brackets.

Comparative analyses of the genomes of *At. ferrivorans* CF27 and six other *Acidithiobacillus* spp., based on groups of orthologous protein clusters (OG), are shown in **Figure 2** and **Supplementary Figure S4**. As shown in the Venn diagram (**Supplementary Figure S4**), 1,399 common OG, accounting for 31.8% of the total proteome, are shared by the seven strains in distinct proportions in each strain: 37.7% for *At. ferrivorans* CF27, 38.6% for *At. ferrivorans* SS3, 40.0% for *At. ferrooxidans* ATCC 23270^T^, 44.8% for *At. ferrooxidans* ATCC 53993, 46.1% for *At. thiooxidans* ATCC 19377^T^, 41.4% for *At. caldus* SM-1 and 50.4% for *At. caldus* ATCC 51756^T^. In addition, the percentages of common OG associated to each species in which the genomes of two strains are available (i.e., *At. ferrivorans, At. ferrooxidans* and *At. caldus*) were 44.9, 57.4, and 57.0%, respectively. These results illustrate the high variability of gene contents in *Acidithiobacillus* genomes and therefore the unique nature of these species. As expected, the *Acidithiobacillus* core genome is mainly involved in essential cellular functions such as DNA replication, translation, amino acid transport and metabolism, central metabolism, as well as in RISCs oxidation. In this core genome, five highly conserved gene families specific to the *Acidithiobacillus* spp. have been recently described (Gonzalez et al., 2016).

The very few OG shared by two to six *Acidithiobacillus* strains, mainly consisted of accessory proteins (**Supplementary Figure S4**). Surprisingly, 19 (0.4%) OG are shared by all *Acidithiobacillus* species, except *At. ferrivorans* CF27, including proteins involved in phosphate transport, as well as an electron-transfer protein (cytochrome *c*552). The common proteins between the two *At. ferrivorans* strains are predicted to be involved in several biological pathways (e.g., cellulose synthesis) as well as regulation, transporters and electron transfer. Not surprisingly, the genes encoding cold-shock proteins (CspE) and trehalose synthase (TreS), that allows the formation of trehalose implicated in a variety of stress response, in particular in cold adaption by thickening the cell cytoplasm to offset ice formation (Jain and Roy, 2009) (**Supplementary Figure S1** and Table S5), were found in both *At. ferrivorans* strains but not in the mesophilic or moderately thermophilic *Acidithiobacillus* species.

As shown in **Figure 2**, proteins specific to each strain mostly consist of transposases and proteins of unknown function. The proportion of *At. ferrivorans* CF27 (24.3% of the overall genome) or SS3 (24.4%) strain-specific proteins is higher when compared to those found for other *Acidithiobacillus* genomes (8.9–20.3%). Among the *At. ferrivorans* CF27 strain-specific proteins, 82.3% of proteins are either hypothetical proteins or proteins of unknown function. In order to identify *At. ferrivorans* CF27 proteins that are absent in other prokaryotic species, we first performed a blast comparison between the set of 947 *At. ferrivorans* CF27-specific proteins and the *nr* prokaryotic database. This resulted in the identification of 434 (11.2% of the overall CF27 genome) proteins only detected in *At. ferrivorans* CF27 (**Figure 2**). The *At. ferrivorans* CF27 specific proteins have in average 91 amino acids (271 aa for the overall genome), an isoelectric point of 8.3 and a codon adaptation index (CAI) between 0.4 and 0.9 (average: 0.7; similar results were obtained for all of the *At. ferrivorans* CF27 proteome). 14.0% of them are predicted to be located in the cell membrane. All specific genes were located within the chromosome, and 30.0% (131 genes) of the *At. ferrivorans* CF27-specific genes within genomic islands. Genes encoding for these *At. ferrivorans* CF27-specific proteins are dispersed or clustered in its genome. Nineteen specific genes clusters were identified (**Figure 2** and Supplementary Table S6) as described in Section Materials and Methods. Seven of the specific gene clusters harbors four or more genes, among which is the tRNA array unit previously described (Cluster 6) (Tran et al., 2015). Eleven specific clusters (Clusters 1, 5–10, 13, 14, 18, 19) are located within genomic islands. Clusters 5 to 10 are located inside the ∼325 Kbp genomic segment containing numerous GIs and carrying the tRNA array unit and the genes involved in flagella formation and chemotaxis described above. Different hypotheses can be proposed to explain the origin and the evolution of these strain-specific genes, including: (i) they were originally widespread in genomes of *Acidithiobacillus* spp., but have been lost in the course of time, except in the case of *At. ferrivorans* CF27; (ii) they were acquired by *At. ferrivorans* CF27 via HGT from an organism which genome is so far unknown; (iii) they were acquired a long time ago and/or diverged quickly to the point that no more similarities could be detected, either by HGT or by gene duplication in the *At. ferrivorans* CF27 genome. The most likely explanation is that the *At. ferrivorans* CF27-specific gene clusters originate from the insertion of mobile genetic elements, since genes predicted to encode phage, transposon or plasmid proteins have been detected in the vicinity of all Clusters, except Cluster 2, described in **Figure 2**, (Supplementary Table S7). These genes include: putative phage assembly proteins (Clusters 3 and 4), reverse transcriptases (Clusters 16 and 17), phage-type recombinase (Cluster 12), phage type endonuclease (Cluster 12), HNH endonucleases (Clusters 8 and 9), relaxase (Cluster 1), integrases (Clusters 12, 13, 16, 18, and 19), resolvases (Clusters 14 and 18), transposases (Clusters 5, 6, 14, 15, and 17), conjugation proteins (Clusters 6, 7, 10, and 11), mobilization protein (Cluster 1), plasmid proteins (Clusters 9, 16, and 18). As more genomic and metagenomic sequences become available, it will be possible to refine our data and to determine which of these hypotheses is more likely.

### Expression of *At. ferrivorans* CF27-Specific Genes

**FIGURE 3:**
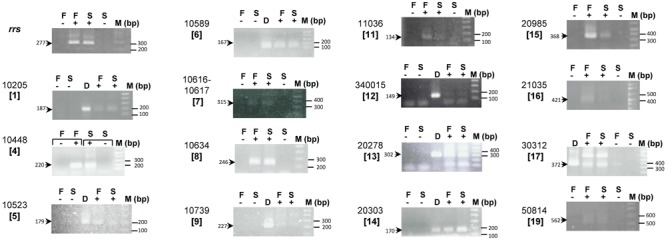
Expression of *At. ferrivorans* CF27-specific genes. RT-PCR experiments were performed on RNA extracted from *At. ferrivorans* CF27 Fe(II)-grown cells (F) or sulfur attached cells (S) without reverse transcriptase (–), with reverse transcriptase (+), and on genomic DNA from *At. ferrivorans* CF27 (D). AFERRI_v2 (or v1) number of each gene is shown with the corresponding cluster number in square brackets. The size of the expected PCR products is indicated. M is the 1 Kbp plus DNA ladder from Invitrogen.

Whether the *At. ferrivorans* CF27-specific genes annotated as ‘hypothetical protein’ or ‘protein of unknown function’ are transcribed or are pseudogenes was investigated further. For this, at least one gene from each *At. ferrivorans* CF27-specific gene cluster was selected, and its transcription when bacteria were grown on either ferrous iron or elemental sulfur was determined by RT-PCR. Attempts to obtain RNA from sulfur-grown planktonic cells were unsuccessful, which was explained by the marked propensity of *At. ferrivorans* CF27 to form macroscopic biofilms in liquid media which results in relatively few planktonic cells when grown in the presence of solid substrates (Blake and Johnson, 2000; Talla et al., 2014). In some clusters, when two genes were transcribed in the same direction and when the intergenic region was less than 40 bp, co-transcription of genes was also analyzed. Four different results were obtained (**Figure 3, Table 1A** and Supplementary Table S8): (i) a single band of expected size; (ii) a band of the expected size together with some non-specific bands (with unexpected sizes); (iii) non-specific bands only; (iv) zero band. No band or non-specific bands have been obtained between two or more genes (Supplementary Table S8) or for four single genes (**Table 1A** and Supplementary Table S8) indicating that these genes were not (co)transcribed under the conditions utilized. A single band with the expected size was detected in 12 cases. It was concluded that the corresponding genes were (co)transcribed in Fe(II)-grown cells and/or sulfur-attached cells (**Table 1A**). When a band of the expected size was obtained together with some non-specific bands, it was amplified by PCR, purified and sequenced. In all the cases, the sequence corresponded to that of the gene under study. Therefore, it was possible to conclude that four additional genes were transcribed in Fe(II)-grown cells and/or in sulfur-attached cells (**Table 1A**). The positive signal obtained with the genes AFERRI_v2_10616 and AFERRI_v2_10617 (Cluster 7) clearly suggests that they are co-transcribed, and therefore should belong to the same operon. It was noted that the AFERRI_v2_10634 gene (Cluster 8), which overlaps the AFERRI_v2_10633 gene, was transcribed while AFERRI_v2_10633 was not, therefore indicating that the true gene is AFERRI_v2_10634. The same conclusion could be drawn from Cluster 12 in which AFERRI_v1_340015, and not AFERRI_v2_20087, was transcribed. Interestingly, three genes were more transcribed in Fe(II)-grown cells than in sulfur-attached cells, while two genes were slightly more expressed in sulfur-attached cells (**Table 1B**). Notably, the Cluster 11 genes are located inside the *cta* locus which was predicted to be involved in cytochrome *aa*_3_ biogenesis and shown to be more transcribed in Fe(II)- than in sulfur-grown cells in *At. ferrooxidans* ATCC 23270^T^ (Quatrini et al., 2009). The other 11 genes seemed to be similarly expressed in both cell growth conditions, as was the 16S rRNA gene. Together these experiments demonstrated that most *At. ferrivorans* CF27-specific genes analyzed were expressed in the prevailing growth conditions of this strain and therefore likely have specific functions. In addition, this not only facilitated refinement of the annotation of the *At. ferrivorans* CF27 genome [e.g., AFERRI_v2_10633/AFERRI_v2_10634 and AFERRI_v1_340015/AFERRI_v2_20087 (Supplementary Table S8)], but also the identification of some specific proteins that could be involved in a particular metabolic pathway (those that are preferentially expressed in one growth condition). For example, genes AFERRI_v2_11036 (Cluster 11), AFERRI_v2_20985 (Cluster 15), AFERRI_v2_21035 (Cluster 16) could play a role during Fe(II) oxidation pathways while AFERRI_v2_10205 (Cluster 1) and AFERRI_v2_20303 (Cluster 14) could either be involved in RISC metabolism or cell attachment (**Table 1B**).

**Table 1:** Expression of *At. ferrivorans* CF27-specific genes.

(A) RT-PCR	

**RT-PCR results**	**Gene ID**

A single band of expected size	AFERRI_v2_10205 (Cluster 1), AFERRI_v2_10448 (Cluster 4), AFERRI_v2_10589 (Cluster 6), AFERRI_v2_10616 and AFERRI_v2_10617 (Cluster 7), AFERRI_v2_10634 (Cluster 8), AFERRI_v2_11036 (Cluster 11), AFERRI_v1_340015 (Cluster 12), AFERRI_v2_20303 (Cluster 14), AFERRI_v2_20985 (Cluster 15), AFERRI_v2_21035 (Cluster 16), AFERRI_v2_50814 (Cluster 19)
A band of the expected size together with non-specific bands	AFERRI_v2_10523 (Cluster 5), AFERRI_v2_10739 (Cluster 9), AFERRI_v2_20278 (Cluster 13), AFERRI_v2_30312 (Cluster 17)
Non-specific bands	AFERRI_v2_10835 (Cluster 10), AFERRI_v2_40075 (Cluster 18)
Zero band	AFERRI_v2_10428 (Cluster 2), AFERRI_v2_10439 (Cluster 3)

(B) Expression in the different growth conditions	

**Expression**	**Gene ID**

Fe(II) > Sulfur-attached cells	AFERRI_v2_11036 (Cluster 11), AFERRI_v2_20985 (Cluster 15), AFERRI_v2_21035 (Cluster 16)
Fe(II) = Sulfur-attached cells	AFERRI_v2_10448 (Cluster 4), AFERRI_v2_10523 (Cluster 5), AFERRI_v2_10589 (Cluster 6), AFERRI_v2_10616 and AFERRI_v2_10617 (Cluster 7), AFERRI_v2_10634 (Cluster 8), AFERRI_v2_10739 (Cluster 9), AFERRI_v2_340015 (Cluster 12), AFERRI_v2_20278 (Cluster 13), AFERRI_v2_30312 (Cluster 17), AFERRI_v2_50814 (Cluster 19)
Fe(II) ≤ Sulfur-attached cells	AFERRI_v2_10205 (Cluster 1), AFERRI_v2_20303 (Cluster 14)

### The Genomes of *At. ferrivorans* CF27 Genome and Other *Acidithiobacillus* spp. Exhibit Significant Chimeric Taxonomic Gene Composition

**FIGURE 4:**
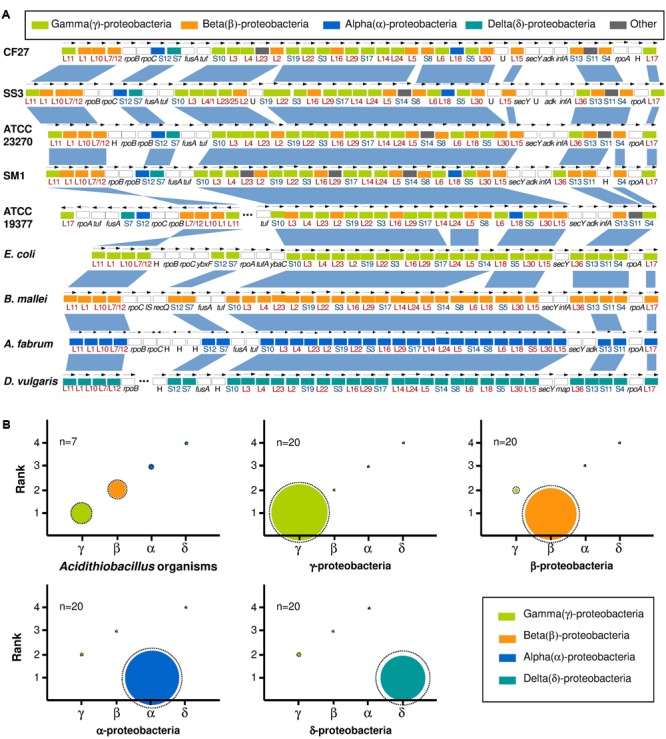
The mosaic gene composition of *Acidithiobacillus* genomes. **(A)** Taxonomic affiliation of ribosomal proteins (colored boxes). The color indicates the closest affiliation of ribosomal proteins with corresponding phyla. Vertical blue bands illustrate syntenic regions between ribosomal proteins. Open bars (boxes) represent other genes. Gray bars (Other) represent other taxonomic classes (except for proteobacterial classes). The strain names are: *B. subtilis, Bacillus subtilis* 168; CF27, *At. ferrivorans* CF27; SS3, *At. ferrivorans* SS3; ATCC 23270, *At. ferrooxidans* ATCC 23270^T^; ATCC 53993, *At. ferrooxidans* 53993; ATCC 19377, *At. thiooxidans* ATCC 19377^T^; SM1, *At. caldus* SM-1; and ATCC 51756, *At. caldus* ATCC 51756^T^; *E. coli, Escherichia coli* K12; *A. fabrum, Agrobacterium fabrum* C58; *B. mallei, Burkholderia mallei* ATCC 23344; *D. vulgaris, Desulfovibrio vulgaris* Hildenborough. **(B)** For each phylum, the relative proportion of taxonomic affiliations is represented by the colored space area (mean value) at each rank. Dashed circles represent the maximal value of relative taxonomic affiliations obtained from Mean ± SD.

Although the Acidithiobacillales were originally assigned to the class Gammaproteobacteria (Garrity et al., 2005), they were more recently transferred to a new class in the proteobacterial phylum, the Acidithiobacillia (Williams and Kelly, 2013). To further understand this phylogenetic classification at the protein level, we first performed a top-scoring blast analysis of ribosomal protein markers (Matte-Tailliez et al., 2002; Chen and Rosen, 2014) from the four *Acidithiobacillus* species, using *Agrobacterium fabrum* C58 (Alphaproteobacteria), *Burkholderia mallei* ATCC 23344 (Betaproteobacteria), *Escherichia coli* K12 (Gammaproteobacteria), and *Desulfovibrio vulgaris* Hildenborough (Deltaproteobacteria) as reference species of their class. As expected, ribosomal proteins from reference species displayed the highest scores with those of their related phyla, which is fully consistent with the taxonomic classification of these bacteria, based on classical 16S rRNA phylogeny or using other phylogenetic markers. Intriguingly, the *At. ferrivorans* CF27 and other *Acidithiobacillus* ribosomal proteins displayed a chimeric nature composition with proteins highly similar to Alpha-, Beta-, Gamma-, and Deltaproteobacteria (**Figure 4A**). Notably, the numbers of ribosomal proteins affiliated to Beta- or Gammaproteobacteria were significantly higher than those of Alpha- or Deltaproteobacteria origins.

To assess whether these observations reflect global genomic features of the *Acidithiobacillus* spp., we further analyzed the whole CDS of these species in comparison with that of 20 representatives from each Alpha-, Beta-, Gamma-, or Deltaproteobacteria classes. As expected, taxonomic affiliations of protein coding sequences from Alpha-, Beta-, Delta-, Gammaproteobacteria reference species were mostly distributed in Alphaproteobacteria (86.1% ± 7.7), Betaproteobacteria (81.5% ± 9.1), Deltaproteobacteria (70.2% ± 15.4), Gammaproteobacteria (89.8% ± 5.4) taxonomic lineages, respectively. Unlike these proteobacterial subgroups, CDS from *Acidithiobacillus* species were mainly assigned to Gammaproteobacteria (31.0% ± 2.2), Betaproteobacteria (27.3% ± 2.1), Alpha-, Deltaproteobacteria (5.7% ± 0.8, 2.7% ± 0.3, respectively), and Firmicutes (2.2% ± 0.4) (**Figure 4B** and Supplementary Table S9). The high proportion and dislocated occurrence of the chimeric regions observed in *Acidithiobacillus* genomes would rule out the possibility of a recent acquisition via HGT from Gamma- and Betaproteobacteria. However, the genes affiliated to Alpha-, Delta-, Epsilonproteobacteria and other phyla which are in much smaller proportion, could have been acquired by HGT during the evolution. These results indicate that *Acidithiobacillus* genomes have a chimeric taxonomic composition (in both ribosomal and whole-genome level) with almost equal proportion of affiliation to Gamma-, and Betaproteobacteria. These chimeric taxonomic patterns for *Acidithiobacillus* genomes strongly support that *Acidithiobacillus* spp. belong to a particular taxonomic class, distinct from other proteobacterial subgroups, and close to the Betaproteobacteria and Gammaproteobacteria classes, as previously proposed (Williams and Kelly, 2013). Similar analyses have been performed in the genomes of Alphaproteobacteria (Esser et al., 2007), and great variation in the data (between 33 and 97%) pointed out a chimeric trait of several alphaproteobacterial genomes. In addition, this strategy, combines to other evolutionary analyses, has been recently used to define a novel group of Proteobacteria, named Etaproteobacteria (Ji et al., 2017). These current data demonstrated, for the first time, that the top-scoring Blast analysis can be useful to define or confirm that a set of organisms belong to a particular taxonomic groups. Therefore, taxonomic affiliations based on ribosomal proteins and whole genome CDS could be used to reflect the phylogenetic trait of an organism or a set of organisms.

## Conclusion

• We have described an updated *At. ferrivorans* CF27 genome assembly, representing substantial improvements to that previously published, with improved gene annotation and gene function assignment.
• Fifty-one genomic islands, in association with some specific features (e.g., biofilm synthesis, tRNA gene unit, chemotaxis and flagella), have been identified.
• *Acidithiobacillus ferrivorans* CF27 exhibits gene determinants involved in cold adaptation and metal(loid) resistance in agreement with the physicochemical characteristics of its isolation site (Hallberg et al., 2010).
• Comparative genome analysis of *At. ferrivorans* CF27 and closely related *Acidithiobacillus* spp. (*At. ferrooxidans, At. thiooxidans, At. caldus* and *At. ferrivorans* SS3) identified high proportions of strain-specific genes in the genomes of *At. ferrivorans* strains CF27 and SS3. Genes such as these may significantly contributed to genome evolution and therefore to adaptation to environmental changes in harsh conditions. Some *At. ferrivorans* CF27-specific genes were shown to be expressed, suggesting functional roles when the bacteria are grown in ferrous iron and sulfur media.
• Our analysis indicates a chimeric origin of *Acidithiobacillus* species of which ribosomal proteins and whole genome coding sequences were mainly affiliated to Gamma- and Betaproteobacteria. The chimeric taxonomic pattern strongly supports the view that *Acidithiobacillus* species belong to a separate taxonomic class within the Proteobacteria.

## Author Contributions

ET and VB conceived and designed the experiments. TT, SM, GM, EP, ZR, HB, BMG, and VB performed the experiments. TT, HB, VB, and ET analyzed the data. TT, SM, GM, EP, ZR, HB, BMG, DBJ, VB and ET contributed to the reagents/materials/analysis tools. TT, DBJ, VB, and ET wrote the paper. All authors read and approved the final manuscript.

## Supplementary Material

The Supplementary Material for this article can be found online at: http://journal.frontiersin.org/article/10.3389/fmicb.2017.01009/full#supplementary-material

FIGURE S1 Trehalose biosynthesis in *Acidithiobacillus ferrivorans* CF27.**(A)** Various pathways for trehalose biosynthesis in *At. ferrivorans* CF27. Functional description of proteins are: TreS, trehalose synthase; GlgX, glycogen debranching protein; TreZ, malto-oligosyltrehalose trehalohydrolase; TreY, malto-oligosyltrehalose synthase. The trehalose operon is shown. **(B)** Synteny map of trehalose biosynthesis genes between *Acidithiobacillus* strains. The upper panel shows *At. ferrivorans* CF27 while the lower panel shows the other *Acidithiobacillus* strains. The genes predicted to be involved in trehalose biosynthesis are boxed. They include *treS* for trehalose synthase, *glgX* for glycogen debranching protein, *treZ* for malto-oligosyltrehalose trehalohydrolase and *treY* for malto-oligosyltrehalose synthase (see Supplementary Table S5 for AFERRI_v2_numbers). The same color tone indicates the same localization.

FIGURE S2 Transition metal and arsenic resistance mechanisms in *Acidithiobacillus* spp.. Organization of *cus* **(A)**, *corA* **(B)**, *mer* **(C)** and *ars* **(D)** operons in *At. ferrivorans* CF27 (see Supplementary Table S5 for AFERRI_v2_numbers) and other *Acidithiobacillus* genomes. The strain names are: CF27, *At. ferrivorans* CF27; SS3, *At. ferrivorans* SS3; ATCC 23270, *At. ferrooxidans* ATCC 23270^T^; ATCC 53993, *At. ferrooxidans* 53993; ATCC 19377, *At. thiooxidans* ATCC 19377^T^; SM-1, *At. caldus* SM-1; and ATCC 51756, *At. caldus* ATCC 51756^T^. The genes and operons are: *cusABCF* involved in copper resistance; *corA* involved in zinc resistance; *merABCDTR* involved in mercury resistance; *arsABCDRH* involved in arsenic resistance; *cyt*, cytochrome *b*_561_; U, protein of unknown function; *X*, heavy metal transport/detoxification protein; *Y*, FAD-dependent pyridine nucleotide-disulfide oxidoreductase; *cag*, glucan 1,4-α-glucosidase; *IS*, ISAfe3 transposase; *MFS*, major facilitator superfamily. Gene synteny is displayed by connecting lines between the orthologous genes. Genes involved in metal(loid) resistance presenting similarities with *At. ferrivorans* CF27 genes are shown in blue (same transcriptional direction) or in green (opposite transcriptional direction). Other genes are illustrated in gray. Operons located in plasmids were boxed in dotted line. **(E)** Copper, zinc, mercury and arsenic resistance mechanisms present in *At. ferrivorans* CF27. Copper resistance components: CopZ, putative cytoplasmic copper chaperone, CopA1 and CopB, copper-exporting P-type ATPases; CusCFBA, copper efflux pump system; Pho84, copper-phosphate complex transporter. Zinc and manganese resistance components: CzcCBA, Zn^2+^ efflux pump system; CzcD, Co^2+^-Zn^2+^-Cd^2+^ efflux protein; CorA, Mg^2+^/Co^2+^/Zn^2+^ transporter protein. Mercury resistance components: MerP, periplasmic mercuric ion binding protein; MerT, mercuric ion transporter; MerA, mercuric reductase. Arsenic resistance components: GlpF, glycerol MIP channel; PstACS, high affinity phosphate transport system; ArsC, arsenate reductase; ArsD, arsenical resistance operon trans-acting repressor and arsenic chaperone; ArsAB, arsenic efflux pump. Gene clusters are represented in gray arrows. OM, Outer Membrane; IM, Inner Membrane; Ec, Extracellular.

FIGURE S3 **(A)** Synteny of chemotaxis and flagellar biosynthesis genes between *Acidithiobacillus* species. The upper 3anel shows the cluster of genes involved in chemotaxis and flagellar biosynthesis in *At. ferrivorans* CF27 (see Supplementary Table S5 for AFERRI_v2_numbers) while the lower panel shows these clusters in the other *Acidithiobacillus* genomes. In between the two panels are given numbers corresponding to the genes with the following functional descriptions: 1, transposase; 2, methyl-accepting chemotaxis sensory transducers; 3, chemotaxis protein (CheV); 4, flavoprotein; 5, signal transduction histidine kinase (CheA); 6, putative chemotaxis phosphatase (CheZ); 7; chemotaxis regulator transmitting signal to flagellar motor component (CheY); 8, flagellar motor rotation protein (MotB); 9, flagellar motor component (MotA); 10, flagellar basal body-associated protein (FliL); 11, putative flagellar export pore protein (FlhA); 12, flagellar biosynthesis protein (FlhB); 13, putative flagellar biosynthetic protein (FliR); 14, putative flagellar biosynthetic protein (FliQ); 15, flagellum-specific ATP synthase (FliI); 16, putative flagellar assembly protein (FliH); 17, putative RNA polymerase sigma factor WhiG (FliA); 18, flagellar biosynthesis protein (FliQ); 19, flagellar biosynthesis protein (FliP); 20, flagellar motor switch protein (FliN); 21, flagellar motor switch protein (FliM); 22, flagellar motor switch protein (FliG); 23, flagellar M-ring protein (FliF); 24, flagellar basal-body component (FliE); 25, flagellin-specific chaperone (FliS); 26, flagellar hook-associated protein (FliD); 27, flagellin protein (FliC); 28, glycosyltransferase; 29, flagellar hook-associated protein (FlgL); 30, flagellar hook-associated protein (FlgK); 31, putative peptidoglycan hydrolase (FlgJ); 32, flagellar basal body P-ring protein (FlgI); 33, flagellar L-ring protein (FlgH); 34, flagellar component of cell-distal portion of basal-body rod (FlgG); 35, flagellar basal-body rod protein (FlgF); 36, flagellar hook protein (FlgE); 37, flagellar hook capping protein (FlgD); 38, flagellar component of cell-proximal portion of basal-body rod (FlgC); 39, flagellar basal body rod protein (FlgB); 40, transcriptional regulator. The genes encoding proteins of unknown function are not indicated. The same color tone indicates the same localization. **(B)** Model of chemotaxis and flagella in *At. ferrivorans* CF27. Flagellar picture was adapted from Liu and Ochman (2007).

FIGURE S4 Venn diagram of orthologous groups (OG) from seven *Acidithiobacillus* genomes. The strain names are: CF27, *At. ferrivorans* CF27; SS3, *At. ferrivorans* SS3; ATCC 23270, *At. ferrooxidans* ATCC 23270^T^; ATCC 53993, *At. ferrooxidans* ATCC 53993; ATCC 19377, *At. thiooxidans* ATCC 19377^T^; SM1, *At. caldus* SM-1; and ATCC 51756, *At. caldus* ATCC 51756^T^. OGs are displayed by the roman numerals (from I to IV). The numbers in brackets indicate the number of genes in each strain and their percentage (compared to the corresponding proteome) are shown in parentheses. Main functional proteins are shown in color boxes and gene cluster is shown in an open bracket.

## Conflict of Interest Statement

The authors declare that the research was conducted in the absence of any commercial or financial relationships that could be construed as a potential conflict of interest.

## Footnotes

1 http://www.neb.com

2 www.beckmancoulter.com

3 https://nanoporetech.com/

4 http://cab.spbu.ru/software/spades

5 www.genoscope.cns.fr

6 https://www.genoscope.cns.fr/agc/microscope

7 http://www.ebi.ac.uk/ena/

8 http://www.pathogenomics.sfu.ca/islandviewer/browse/

9 http://emboss.sourceforge.net

10 ftp://ftp.ncbi.nih.gov/

## References

[B1] Aiba H. Adhya S. De Crombrugghe B. (1981). Evidence for two functional *gal* promoters in intact *Escherichia coli* cells. *J. Biol. Chem.* 256 11905–11910. 6271763

[B2] Amouric A. Brochier-Armanet C. Johnson D. B. Bonnefoy V. Hallberg K. B. (2011). Phylogenetic and genetic variation among Fe(II)-oxidizing acidithiobacilli supports the view that these comprise multiple species with different ferrous iron oxidation pathways. *Microbiology* 157 111–122. 10.1099/mic.0.044537-0 20884692

[B3] Barahona S. Dorador C. Zhang R. Aguilar P. Sand W. Vera M. (2014). Isolation and characterization of a novel *Acidithiobacillus ferrivorans* strain from the Chilean Altiplano: attachment and biofilm formation on pyrite at low temperature. *Res. Microbiol.* 165 782–793. 10.1016/j.resmic.2014.07.015 25111023

[B4] Blake R. Johnson D. B. (2000). “Phylogenetic and biochemical diversity among acidophilic bacteria that respire on iron,” in *Environmental Microbe-Metal Interactions* ed. Lovley D. E. (Washington, DC: ASM Press) 53–78. 10.1128/9781555818098.ch3

[B5] Chen J. Rosen B. P. (2014). Biosensors for inorganic and organic arsenicals. *Biosensors* 4 494–512. 10.3390/bios4040494 25587436 PMC4287715

[B6] Christel S. Fridlund J. Watkin E. L. Dopson M. (2016). *Acidithiobacillus ferrivorans* SS3 presents little RNA transcript response related to cold stress during growth at 8 degrees C suggesting it is a eurypsychrophile. *Extremophiles* 20 903–913. 10.1007/s00792-016-0882-2 27783177 PMC5085989

[B7] Dhillon B. K. Laird M. R. Shay J. A. Winsor G. L. Lo R. Nizam F. (2015). IslandViewer 3: more flexible, interactive genomic island discovery, visualization and analysis. *Nucleic Acids Res.* 43 W104–W108. 10.1093/nar/gkv401 25916842 PMC4489224

[B8] Dopson M. (2016). “Physiological and phylogenetic diversity of acidophilic bacteria,” in *Acidophiles: Life in Extremely Acidic Environments* eds Quatrini R. Johnson D. B. (Poole: Caister Academic Press) 79–82. 10.21775/9781910190333.05

[B9] Dopson M. Holmes D. S. (2014). Metal resistance in acidophilic microorganisms and its significance for biotechnologies. *Appl. Microbiol. Biotechnol.* 98 8133–8144. 10.1007/s00253-014-5982-2 25104030

[B10] Esser C. Martin W. Dagan T. (2007). The origin of mitochondria in light of a fluid prokaryotic chromosome model. *Biol. Lett.* 3 180–184. 10.1098/rsbl.2006.0582 17251118 PMC2375920

[B11] Falagán C. Johnson D. B. (2016). *Acidithiobacillus ferriphilus* sp. nov., a facultatively anaerobic iron- and sulfur-metabolizing extreme acidophile. *Int. J. Syst. Evol. Microbiol.* 66 206–211. 10.1099/ijsem.0.000698 26498321 PMC4806540

[B12] Federhen S. (2012). The NCBI Taxonomy database. *Nucleic Acids Res.* 40 D136–D143. 10.1093/nar/gkr1178 22139910 PMC3245000

[B13] Garrity G. M. Bell J. A. Lilburn T. (2005). “Order II. Acidithiobacillales ord. nov,” in *Bergey’s Manual of Systematic Bacteriology* eds Brenner D. Krieg N. Staley J. Garrity G. (New York, NY: Springer) 10.1016/B978-012373944-5.00154-1

[B14] Gonzalez C. Lazcano M. Valdes J. Holmes D. S. (2016). Bioinformatic analyses of unique (orphan) core genes of the genus *Acidithiobacillus*: functional inferences and use as molecular probes for genomic and metagenomic/transcriptomic interrogation. *Front. Microbiol.* 7:2035 10.3389/fmicb.2016.02035 PMC5186765 28082953

[B15] Hallberg K. B. Gonzalez-Toril E. Johnson D. B. (2010). *Acidithiobacillus ferrivorans*, sp. nov.; facultatively anaerobic, psychrotolerant iron-, and sulfur-oxidizing acidophiles isolated from metal mine-impacted environments. *Extremophiles* 14 9–19. 10.1007/s00792-009-0282-y 19787416

[B16] Hedrich S. Johnson D. B. (2013). *Acidithiobacillus ferridurans* sp. nov., an acidophilic iron-, sulfur- and hydrogen-metabolizing chemolithotrophic gammaproteobacterium. *Int. J. Syst. Evol. Microbiol.* 63 4018–4025. 10.1099/ijs.0.049759-0 23710060

[B17] Hsiao W. W. Ung K. Aeschliman D. Bryan J. Finlay B. B. Brinkman F. S. (2005). Evidence of a large novel gene pool associated with prokaryotic genomic islands. *PLoS Genet.* 1:e62 10.1371/journal.pgen.0010062 PMC1285063 16299586

[B18] Jain N. K. Roy I. (2009). Effect of trehalose on protein structure. *Protein Sci.* 18 24–36. 10.1002/pro.3 19177348 PMC2708026

[B19] Jerez C. A. (2009). “Metal extraction and biomining,” in *The Desk Encyclopedia of Microbiology* 3rd Edn ed. Schaechter M. (Oxford: Elsevier) 10.1016/B978-012373944-5.00154-1

[B20] Ji B. Zhang S. D. Zhang W. J. Rouy Z. Alberto F. Santini C. L. (2017). The chimeric nature of the genomes of marine magnetotactic coccoid-ovoid bacteria defines a novel group of *Proteobacteria*. *Environ. Microbiol.* 19 1103–1119. 10.1111/1462-2920.13637 27902881

[B21] Johnson D. B. (2014). Biomining-biotechnologies for extracting and recovering metals from ores and waste materials. *Curr. Opin. Biotechnol.* 30 24–31. 10.1016/j.copbio.2014.04.008 24794631

[B22] Kelly D. P. Wood A. P. (2000). Reclassification of some species of *Thiobacillus* to the newly designated genera *Acidithiobacillus* gen. nov., *Halothiobacillus* gen. nov. and *Thermithiobacillus* gen. nov. *Int. J. Syst. Evol. Microbiol.* 50(Pt 2) 511–516. 10.1099/00207713-50-2-511 10758854

[B23] Krzywinski M. Schein J. Birol I. Connors J. Gascoyne R. Horsman D. (2009). Circos: an information aesthetic for comparative genomics. *Genome Res.* 19 1639–1645. 10.1101/gr.092759.109 19541911 PMC2752132

[B24] Kupka D. Liljeqvist M. Nurmi P. Puhakka J. A. Tuovinen O. H. Dopson M. (2009). Oxidation of elemental sulfur, tetrathionate and ferrous iron by the psychrotolerant *Acidithiobacillus* strain SS3. *Res. Microbiol.* 160 767–774. 10.1016/j.resmic.2009.08.022 19782750

[B25] Kupka D. Rzhepishevska O. I. Dopson M. Lindstrom E. B. Karnachuk O. V. Tuovinen O. H. (2007). Bacterial oxidation of ferrous iron at low temperatures. *Biotechnol. Bioeng.* 97 1470–1478. 10.1002/bit.21371 17304566

[B26] Langille M. G. Hsiao W. W. Brinkman F. S. (2008). Evaluation of genomic island predictors using a comparative genomics approach. *BMC Bioinformatics* 9:329 10.1186/1471-2105-9-329 PMC2518932 18680607

[B27] Levican G. Katz A. Valdès J. Quatrini R. Holmes D. Orellana O. (2009). A 300 kpb genome segment, including a complete set of tRNA genes, is dispensable for *Acidithiobacillus ferrooxidans*. *Adv. Mater. Res.* 71–73 187–190. 10.4028/www.scientific.net/AMR.71-73.187

[B28] Li L. Stoeckert C. J. Jr. Roos D. S. (2003). OrthoMCL: identification of ortholog groups for eukaryotic genomes. *Genome Res.* 13 2178–2189. 10.1101/gr.1224503 12952885 PMC403725

[B29] Liljeqvist M. Ossandon F. J. Gonzalez C. Rajan S. Stell A. Valdes J. (2015). Metagenomic analysis reveals adaptations to a cold-adapted lifestyle in a low-temperature acid mine drainage stream. *FEMS Microbiol. Ecol.* 91:fiv011 10.1093/femsec/fiv011 25764459

[B30] Liljeqvist M. Valdes J. Holmes D. S. Dopson M. (2011). Draft genome of the psychrotolerant acidophile *Acidithiobacillus ferrivorans* SS3. *J. Bacteriol.* 193 4304–4305. 10.1128/JB.05373-11 21705598 PMC3147677

[B31] Liu R. Ochman H. (2007). Stepwise formation of the bacterial flagellar system. *Proc. Natl. Acad. Sci. U.S.A.* 104 7116–7121. 10.1073/pnas.0700266104 17438286 PMC1852327

[B32] Mamani S. Moinier D. Denis Y. Soulere L. Queneau Y. Talla E. (2016). Insights into the quorum sensing regulon of the acidophilic *Acidithiobacillus ferrooxidans* revealed by transcriptomic in the presence of an acyl homoserine lactone superagonist analog. *Front. Microbiol.* 7:1365 10.3389/fmicb.2016.01365 PMC5021923 27683573

[B33] Matte-Tailliez O. Brochier C. Forterre P. Philippe H. (2002). Archaeal phylogeny based on ribosomal proteins. *Mol. Biol. Evol.* 19 631–639. 10.1093/oxfordjournals.molbev.a004122 11961097

[B34] Nancucheo I. Rowe O. F. Hedrich S. Johnson D. B. (2016). Solid and liquid media for isolating and cultivating acidophilic and acid-tolerant sulfate-reducing bacteria. *FEMS Microbiol. Lett.* 363:fnw083 10.1093/femsle/fnw083 27036143

[B35] Nitschke W. Bonnefoy V. (2016). “Energy acquisition in low pH environments,” in *Acidophiles: Life in Extremely Acidic Environments* eds Quatrini R. Johnson D. B. (Poole: Caister Academic Press) 19–48. 10.21775/9781910190333

[B36] Nuñez H. Covarrubias P. C. Moya-Beltran A. Issotta F. Atavales J. Acuna L. G. (2016). Detection, identification and typing of *Acidithiobacillus* species and strains: a review. *Res. Microbiol.* 167 555–567. 10.1016/j.resmic.2016.05.006 27288569

[B37] Nuñez H. Moya-Beltrán A. Covarrubias P. C. Issotta F. Cardenas J. P. Gonzalez M. (2017). Molecular systematics of the genus *Acidithiobacillus*: insights into the phylogenetic structure and diversification of the taxon. *Front. Microbiol.* 8:30 10.3389/fmicb.2017.00030 PMC5243848 28154559

[B38] Osorio H. Mangold S. Denis Y. Nancucheo I. Esparza M. Johnson D. B. (2013). Anaerobic sulfur metabolism coupled to dissimilatory iron reduction in the extremophile *Acidithiobacillus ferrooxidans*. *Appl. Environ. Microbiol.* 79 2172–2181. 10.1128/AEM.03057-12 23354702 PMC3623243

[B39] Quatrini R. Appia-Ayme C. Denis Y. Jedlicki E. Holmes D. S. Bonnefoy V. (2009). Extending the models for iron and sulfur oxidation in the extreme acidophile *Acidithiobacillus ferrooxidans*. *BMC Genomics* 10:394 10.1186/1471-2164-10-394 PMC2754497 19703284

[B40] Talla E. Hedrich S. Mangenot S. Ji B. Johnson D. B. Barbe V. (2014). Insights into the pathways of iron- and sulfur-oxidation, and biofilm formation from the chemolithotrophic acidophile *Acidithiobacillus ferrivorans* CF27. *Res. Microbiol.* 165 753–760. 10.1016/j.resmic.2014.08.002 25154051

[B41] Tran T. T. Belahbib H. Bonnefoy V. Talla E. (2015). A comprehensive tRNA genomic survey unravels the evolutionary history of tRNA arrays in prokaryotes. *Genome Biol. Evol.* 8 282–295. 10.1093/gbe/evv254 26710853 PMC4758250

[B42] Valdes J. Ossandon F. Quatrini R. Dopson M. Holmes D. S. (2011). Draft genome sequence of the extremely acidophilic biomining bacterium *Acidithiobacillus thiooxidans* ATCC 19377 provides insights into the evolution of the *Acidithiobacillus* genus. *J. Bacteriol.* 193 7003–7004. 10.1128/JB.06281-11 22123759 PMC3232857

[B43] Valdes J. Pedroso I. Quatrini R. Dodson R. J. Tettelin H. Blake R. (2008). *Acidithiobacillus ferrooxidans* metabolism: from genome sequence to industrial applications. *BMC Genomics* 9:597 10.1186/1471-2164-9-597 PMC2621215 19077236

[B44] Valdes J. Quatrini R. Hallberg K. Dopson M. Valenzuela P. D. Holmes D. S. (2009). Draft genome sequence of the extremely acidophilic bacterium *Acidithiobacillus caldus* ATCC 51756 reveals metabolic versatility in the genus *Acidithiobacillus*. *J. Bacteriol.* 191 5877–5878. 10.1128/JB.00843-09 19617360 PMC2737959

[B45] Vallenet D. Calteau A. Cruveiller S. Gachet M. Lajus A. Josso A. (2017). MicroScope in 2017: an expanding and evolving integrated resource for community expertise of microbial genomes. *Nucleic Acids Res.* 45 D517–D528. 10.1093/nar/gkw1101 27899624 PMC5210572

[B46] Waack S. Keller O. Asper R. Brodag T. Damm C. Fricke W. F. (2006). Score-based prediction of genomic islands in prokaryotic genomes using hidden Markov models. *BMC Bioinformatics* 7:142 10.1186/1471-2105-7-142 PMC1489950 16542435

[B47] Williams K. P. Kelly D. P. (2013). Proposal for a new class within the phylum *Proteobacteria, Acidithiobacillia* classis nov., with the type order *Acidithiobacillales*, and emended description of the class *Gammaproteobacteria*. *Int. J. Syst. Evol. Microbiol.* 63 2901–2906. 10.1099/ijs.0.049270-0 23334881

[B48] You X. Y. Guo X. Zheng H. J. Zhang M. J. Liu L. J. Zhu Y. Q. (2011). Unraveling the *Acidithiobacillus caldus* complete genome and its central metabolisms for carbon assimilation. *J. Genet. Genomics* 38 243–252. 10.1016/j.jgg.2011.04.006 21703548

[B49] Zhaxybayeva O. Swithers K. S. Lapierre P. Fournier G. P. Bickhart D. M. Deboy R. T. (2009). On the chimeric nature, thermophilic origin, and phylogenetic placement of the Thermotogales. *Proc. Natl. Acad. Sci. U.S.A.* 106 5865–5870. 10.1073/pnas.0901260106 19307556 PMC2667022

