# Supplementary material for: Comparative Genome Analysis Provides Insights into Both the Lifestyle of Acidithiobacillus ferrivorans Strain CF27 and the Chimeric Nature of the Iron-Oxidizing Acidithiobacilli Genomes
Source: Front Microbiol. 2017 Jun 13;8:1009. doi: 10.3389/fmicb.2017.01009 (PMC5468388; doi:10.3389/fmicb.2017.01009)
Supplement: FIGURE S1 — Trehalose biosynthesis in Acidithiobacillus ferrivorans CF27.(A) Various pathways for trehalose biosynthesis in At. ferrivorans CF27. Functional description of proteins are: TreS, trehalose synthase; GlgX, glycogen debranching protein; TreZ, malto-oligosyltrehalose trehalohydrolase; TreY, malto-oligosyltrehalose synthase. The trehalose operon is shown. (B) Synteny map of trehalose biosynthesis genes between Acidithiobacillus strains. The upper panel shows At. ferrivorans CF27 while the lower panel shows the other Acidithiobacillus strains. The genes predicted to be involved in trehalose biosynthesis are boxed. They include treS for trehalose synthase, glgX for glycogen debranching protein, treZ for malto-oligosyltrehalose trehalohydrolase and treY for malto-oligosyltrehalose synthase (see Supplementary Table S5 for AFERRI_v2_numbers). The same color tone indicates the same localization. [file Data_Sheet_2.pdf]

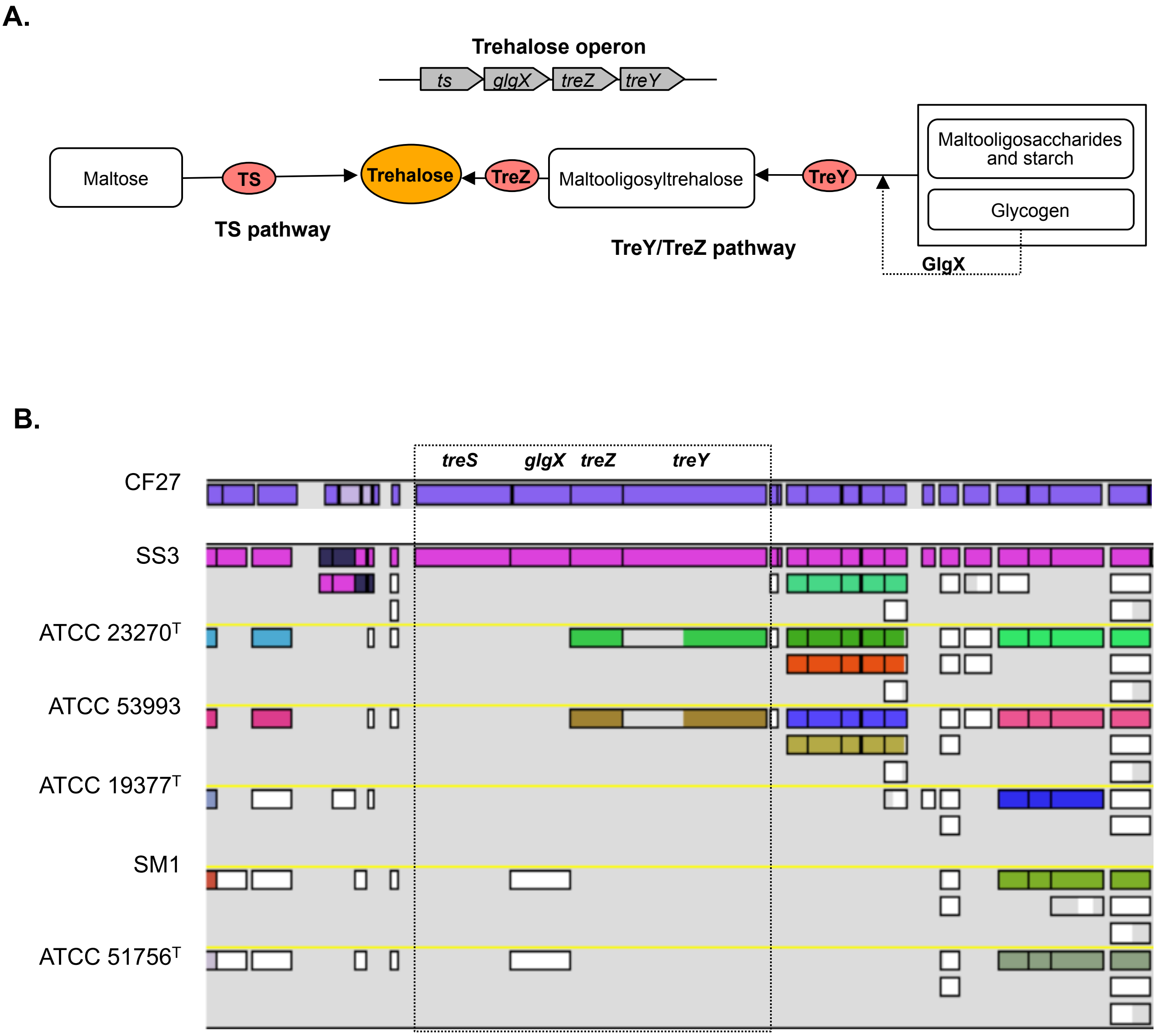

Figure S1. Trehalose biosynthesis in *Acidithiobacillus ferrivorans* CF27. (A) Various pathways for trehalose biosynthesis in *At. ferrivorans* CF27. Functional description of proteins are: TreS, trehalose synthase; GlgX, glycogen debranching protein; TreZ, malto-oligosyltrehalose trehalohydrolase; TreY, malto-oligosyltrehalose synthase. The trehalose operon is shown. **(B)** Synteny map of trehalose biosynthesis genes between *Acidithiobacillus* strains. The upper panel shows *At. ferrivorans* CF27 while the lower panel shows the other *Acidithiobacillus* strains. The genes predicted to be involved in trehalose biosynthesis are boxed. They include *ts* for trehalose synthase, *glgX* for glycogen debranching protein, *treZ* for malto-oligosyltrehalose trehalohydrolase and *treY* for malto-oligosyltrehalose synthase (see **Supplementary Table S5** for AFERRI_v2_ numbers). The same color tone indicates the same localization.

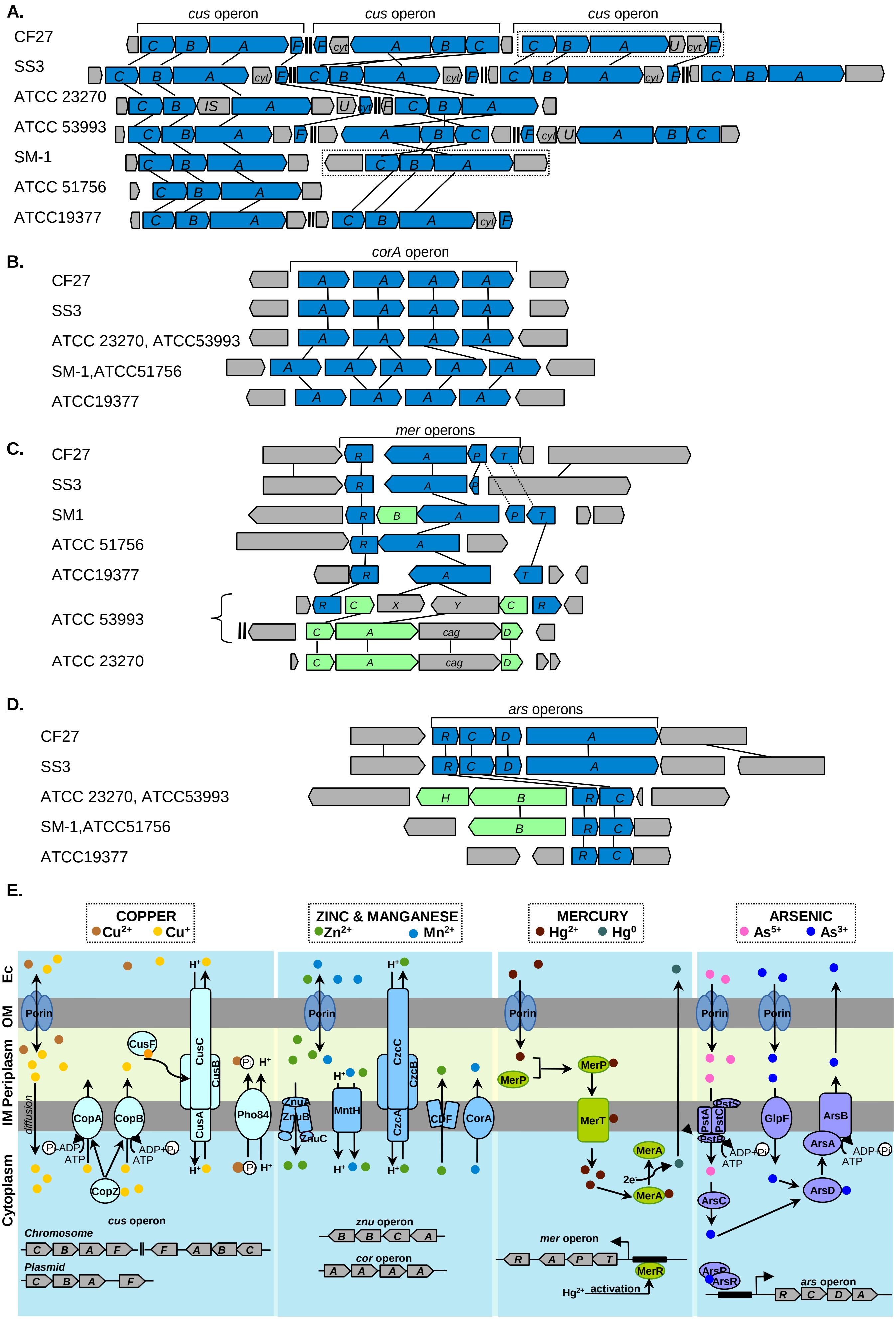

A.
cus operon
cus operon
cus operon
CF27
SS3
ATCC 23270
ATCC 53993
SM-1
ATCC 51756
ATCC19377
B.
corA operon
CF27
SS3
ATCC 23270, ATCC53993
SM-1,ATCC51756
ATCC19377
C.
mer operons
CF27
SS3
SM1
ATCC 51756
ATCC19377
ATCC 53993
ATCC 23270
D.
ars operons
CF27
SS3
ATCC 23270, ATCC53993
SM-1,ATCC51756
ATCC19377
E.
COPPER
Cu2+ Cu+
ZINC & MANGANESE
Zn2+ Mn2+
MERCURY
Hg2+ Hg0
ARSENIC
As5+ As3+
Ec
OM
Periplasm
IM
Cytoplasm
diffusion
Porin
CusF
CusC
CusB
CusA
CopA
CopB
CopZ
Pho84
ZnuA
ZnuB
ZnuC
MntH
CzcC
CzcB
CzcA
CDF
CorA
MerP
MerT
MerA
MerR
PstS
PstA
PstC
PstB
GlpF
ArsB
ArsA
ArsD
ArsC
ArsR
ADP+Pi
ATP
cus operon
Chromosome
Plasmid
znu operon
cor operon
mer operon
Hg2+ activation
ars operon

Figure S2. Transition metal and arsenic resistance mechanisms in *Acidithiobacillus* spp.. Organization of *cus* **(A),** *corA* **(B),** *mer* **(C)** and *ars* **(D)** operons in *At. ferrivorans* CF27 (see **Supplementary Table S5** for AFERRI_v2_numbers) and other *Acidithiobacillus* genomes. The strain names are: CF27, *At. ferrivorans* CF27; SS3, *At. ferrivorans* SS3; ATCC 23270, *At. ferrooxidans* ATCC 23270^T; ATCC 53993, *At. ferrooxidans* 53993; ATCC 19377, *At. thiooxidans* ATCC 19377^T; SM-1, *At. caldus* SM-1; and ATCC 51756, *At. caldus* ATCC 51756^T. The genes and operons are: *cusABCF* involved in copper resistance; *corA* involved in zinc resistance; *merABCDTR* involved in mercury resistance; *arsABCDRH* involved in arsenic resistance; *cyt*, cytochrome b_{561}; U, protein of unknown function; *X*, heavy metal transport/detoxification protein; *Y*, FAD-dependent pyridine nucleotide-disulfide oxidoreductase; *cag*, glucan 1,4-a-glucosidase; *IS*, ISAfe3 transposase; *MFS*, major facilitator superfamily. Gene synteny is displayed by connecting lines between the orthologous genes. Genes involved in metal(loid) resistance presenting similarities with *At. ferrivoran*s CF27 genes are shown in blue (same transcriptional direction) or in green (opposite transcriptional direction). Other genes are illustrated in grey. Operons located in plasmids were boxed in dotted line. (**E)** Copper, zinc, mercury and arsenic resistance mechanisms present in *At. ferrivorans* CF27. Copper resistance components: CopZ, putative cytoplasmic copper chaperone, CopA1 and CopB, copper-exporting P-type ATPases; CusCFBA, copper efflux pump system; Pho84, copper-phosphate complex transporter. Zinc and manganese resistance components: CzcCBA, Zn^{2+} efflux pump system; CzcD, Co^{2+}-Zn^{2+}-Cd^{2+} efflux protein; CorA, Mg^{2+}/Co^{2+}/Zn^{2+} transporter protein. Mercury resistance components: MerP, periplasmic mercuric ion binding protein; MerT, mercuric ion transporter; MerA, mercuric reductase. Arsenic resistance components: GlpF, glycerol MIP channel; PstACS, high affinity phosphate transport system; ArsC, arsenate reductase; ArsD, arsenical resistance operon trans-acting repressor and arsenic chaperone; ArsAB, arsenic efflux pump. Gene clusters are represented in grey arrows. OM, Outer Membrane; IM, Inner Membrane; Ec, Extracellular.

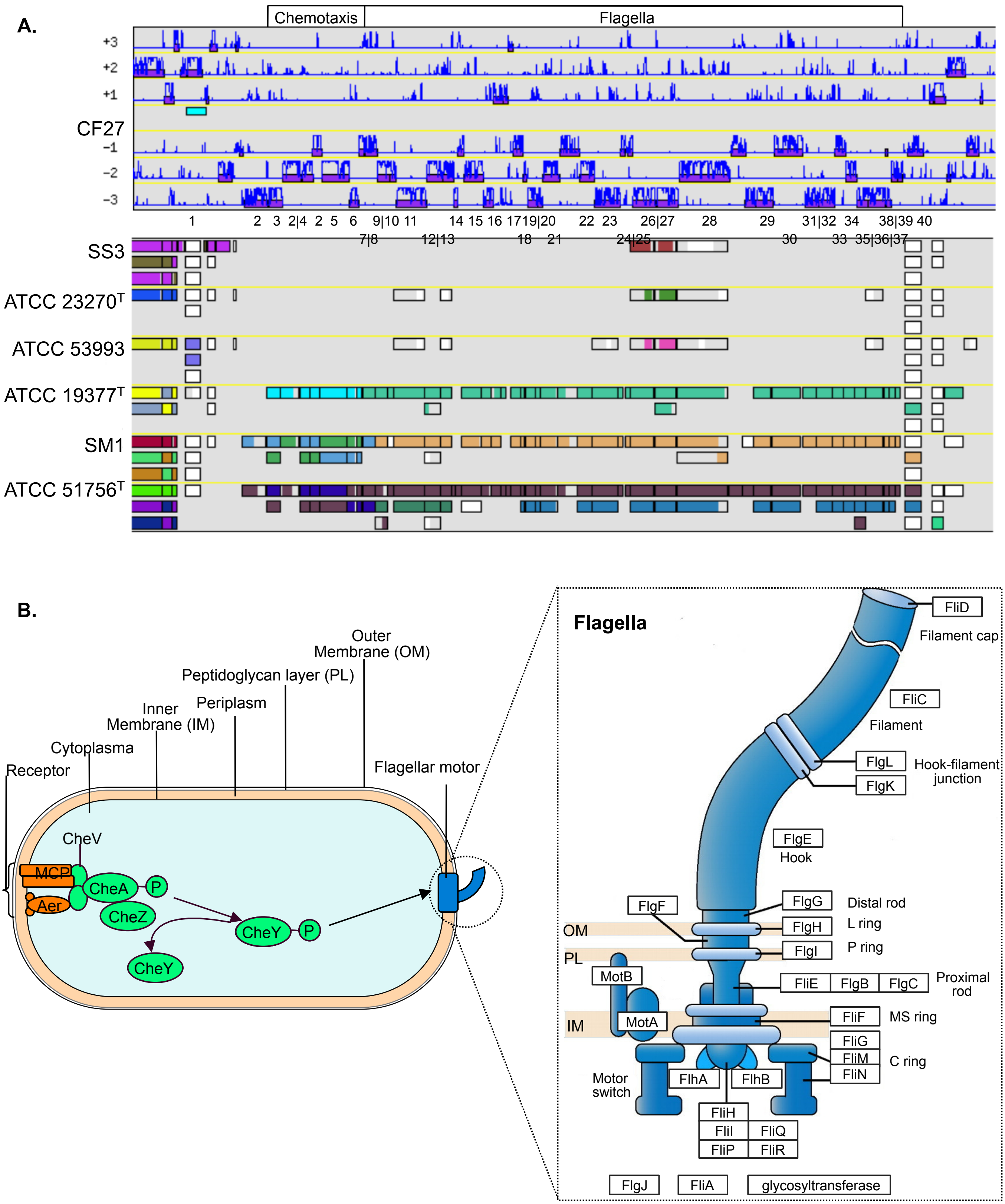
A.
Chemotaxis
Flagella
+3
+2
+1
CF27
−1
−2
−3
1 2 3 2|4 2 5 6 7|8 9|10 11 12|13 14 15 16 17 18 19|20 21 22 23 24|25 26|27 28 29 30 31|32 33 34 35|36|37 38|39 40
SS3
ATCC 23270T
ATCC 53993
ATCC 19377T
SM1
ATCC 51756T
B.
Receptor
Cytoplasma
Inner Membrane (IM)
Periplasm
Peptidoglycan layer (PL)
Outer Membrane (OM)
Flagellar motor
CheV
MCP
Aer
CheA
P
CheZ
CheY
P
CheY
Flagella
FliD
Filament cap
FliC
Filament
FlgL
FlgK
Hook-filament junction
FlgE
Hook
FlgF
FlgG
Distal rod
FlgH
L ring
FlgI
P ring
OM
PL
MotB
FliE
FlgB
FlgC
Proximal rod
IM
MotA
FliF
MS ring
FliG
FliM
FliN
C ring
Motor switch
FlhA
FlhB
FliH
FliI
FliQ
FliP
FliR
FlgJ
FliA
glycosyltransferase

Figure S3. A. **Synteny of chemotaxis and flagellar biosynthesis genes between *Acidithiobacillus* species.** The upper panel shows the cluster of genes involved in chemotaxis and flagellar biosynthesis in *At. ferrivorans* CF27 (see **Supplementary Table S5** for AFERRI_v2_numbers) while the lower panel shows these clusters in the other *Acidithiobacillus* genomes. In between the two panels are given numbers corresponding to the genes with the following functional descriptions: 1, transposase; 2, methyl-accepting chemotaxis sensory transducers; 3, chemotaxis protein (CheV); 4, flavoprotein; 5, signal transduction histidine kinase (CheA); 6, putative chemotaxis phosphatase (CheZ); 7; chemotaxis regulator transmitting signal to flagellar motor component (CheY); 8, flagellar motor rotation protein (MotB); 9, flagellar motor component (MotA); 10, flagellar basal body-associated protein (FliL); 11, putative flagellar export pore protein (FlhA); 12, flagellar biosynthesis protein (FlhB); 13, putative flagellar biosynthetic protein (FliR); 14, putative flagellar biosynthetic protein (FliQ); 15, flagellum-specific ATP synthase (FliI); 16, putative flagellar assembly protein (FliH); 17, putative RNA polymerase sigma factor WhiG (FliA); 18, flagellar biosynthesis protein (FliQ); 19, flagellar biosynthesis protein (FliP); 20, flagellar motor switch protein (FliN); 21, flagellar motor switch protein (FliM); 22, flagellar motor switch protein (FliG); 23, flagellar M-ring protein (FliF); 24, flagellar basal-body component (FliE); 25, flagellin-specific chaperone (FliS); 26, flagellar hook-associated protein (FliD); 27, flagellin protein (FliC); 28, glycosyltransferase; 29, flagellar hook-associated protein (FlgL); 30, flagellar hook-associated protein (FlgK); 31, putative peptidoglycan hydrolase (FlgJ); 32, flagellar basal body P-ring protein (FlgI); 33, flagellar L-ring protein (FlgH); 34, flagellar component of cell-distal portion of basal-body rod (FlgG); 35, flagellar basal-body rod protein (FlgF); 36, flagellar hook protein (FlgE); 37, flagellar hook capping protein (FlgD); 38, flagellar component of cell-proximal portion of basal-body rod (FlgC); 39, flagellar basal body rod protein (FlgB); 40, transcriptional regulator. The genes encoding proteins of unknown function are not indicated. The same colour tone indicates the same localization. **B. Model of chemotaxis and flagella in *At. ferrivorans* CF27.** Flagellar picture was adapted from Liu and Ochaman (2007).

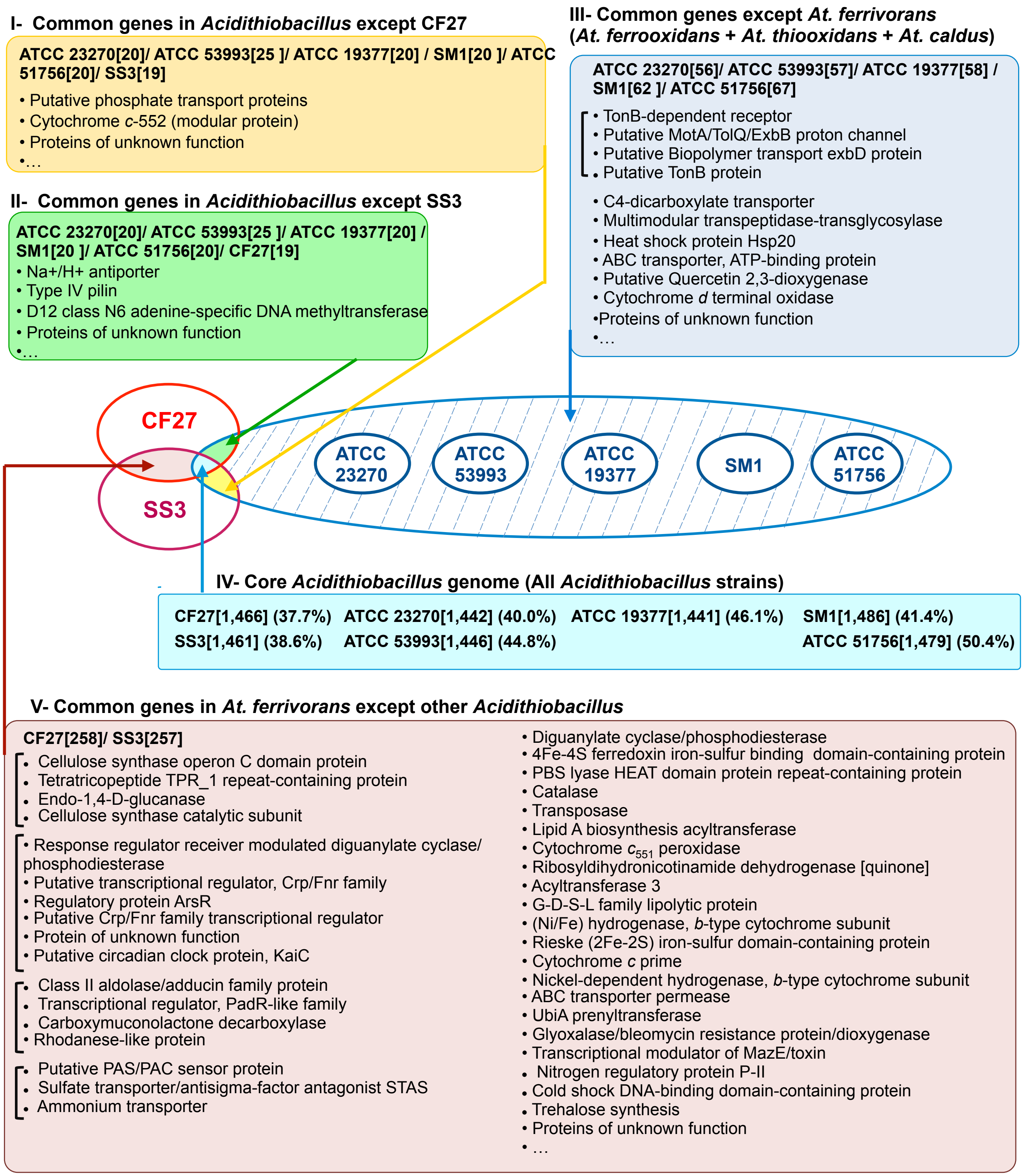

Figure S4. **Venn diagram of orthologous groups (OG) from seven *Acidithiobacillus* genomes**. The strain names are: CF27, *At. ferrivorans* CF27; SS3, *At. ferrivorans* SS3; ATCC 23270, *At. ferrooxidans* ATCC 23270^{T}; ATCC 53993, *At. ferrooxidans* ATCC 53993; ATCC 19377, *At. thiooxidans* ATCC 19377^{T}; SM1, *At. caldus* SM-1; and ATCC 51756, *At. caldus* ATCC 51756^{T}. OGs are displayed by the roman numerals (from I to IV). The numbers in brackets indicate the number of genes in each strain and their percentage (compared to the corresponding proteome) are shown in parentheses. Main functional proteins are shown in colour boxes and gene cluster is shown in an open bracket.
