# Supplementary material for: Comparative Genome Analysis Provides Insights into Both the Lifestyle of Acidithiobacillus ferrivorans Strain CF27 and the Chimeric Nature of the Iron-Oxidizing Acidithiobacilli Genomes
Source: Front Microbiol. 2017 Jun 13;8:1009. doi: 10.3389/fmicb.2017.01009 (PMC5468388; doi:10.3389/fmicb.2017.01009)
Supplement: Supplementary file 2 [file Data_Sheet_1.PDF]

Supplementary Table S1. List of primers used in RT-PCR experiments for amplification of specific genes of *At. ferrivorans* CF27.

Cluster	Reverse oligo name	Reverse oligo sequence	Forward oligo name	Forward oligo sequence	Amplicon size (bp)
1	40163-R	CCATCTGTTCTGGTGACA	40162-F	ACAAGCTCTGGACAAACG	446
	40163-R	CCATCTGTTCTGGTGACA	40163-F	GCGGAAATCTGGAAAAAC	187
2	400155-R	CCGTGTCGTAAAACGT	400152-F	GTTGTCCCTTCCGATTCT	1529
	10429-R	ACGACGATTACGTCGAAC	10428-F	TCTGCCTTTCTTCTGTCG	247
	10428-R	CACCAGACACCCCCTAA	10428-F	TCTGCCTTTCTTCTGTCG	205
3	10439-R	GCCATAACCAGCACAAAG	10436-F	GATGATCGCGCTAAAGG	403
	10439-R	GCCATAACCAGCACAAAG	10439-F	ACGAAAAACAAAGCAAGC	180
4	10448-R	CCAGCTGACAATTCTTGG	10448-F	AGCATTGAGAAGCGACTG	220
5	10523-R	CGGGTTGTCCGAGTAGA	10523-F	GAGCCCGAAAATGTCCT	179
6	10589-R	ACTGCGGTACCCACTCTAC	10589-F	CGAATCACTGGTGGACAT	167
7	400332-R	CAGGGCATACACCTATCG	400335-F	AGGAGCAGGACGTAAAGC	825
	400333-R	CACCGCGTCAGAAAGTAG	400335-F	AGGAGCAGGACGTAAAGC	568
	400333-R	CACCGCGTCAGAAAGTAG	10617-F	GTTTCGTGCTGGTGATGT	315
8	400349-R	ATATGTCACCCGTTGTCG	400350-F	CTCCGAAGCCAATACTGA	293
	400350-R	TTCTGCTTGCGTTTCTG	400350-F	CTCCGAAGCCAATACTGA	164
	10634-R	GAGCATCTGATTCGTTGG	10634-F	CCACGATGTTGAATTGGT	246
9	400460-R	GCCTTCCGGTATTTTCTC	400460-F	ATTCTTACACGCCACACC	227
10	10835-R	GCTGAGATGGATGTACGC	10835-F	TCCATTACCAGCCATCAC	355
11	50032-R	TCCAATAGACCCAGAGGAC	50032-F	ACTTTATGCTGGGCCACT	134
12	340018-R	AAGGTGCGTCATCTTCAG	340017-F	AATGACGTCTTCGCTCTG	361
	340017-R	TCAGCGTCAATTTTCTCC	340017-F	AATGACGTCTTCGCTCTG	227
	340015-R	AAATAAACGCGCCATGTGA	340015-R	GGGGCGGTACGTTTTACA	149
13	20278-R	TCGTCATATCCGCTCCA	20278-F	AATACCGAGCGCACCAT	302
14	20303-R	CGGTCAAGCTCCATAAGA	20303-F	CTGAGTGCCGGTGAGTT	170
15	560073-R	GGGGTCCACCTCGTAATA	560073-F	GCCAGCTTACTCGTCAGA	368
16	21035-R	TTGAACCAGGTGGAGATG	21035-F	ACGGGATGTCCTTTGAAC	421
17	30312-R	CTTCTCGCGTAATCTCCA	30312-F	GCTTCTTGCTGGTTCTCA	372
18	40075-R	CTTGTTCATGCTGCTTCC	40075-F	TACCACTCTGGCCTGTTC	306
19	530146-R	AGATGAGCACGAGAATGC	530146-F	GCTGGAGTTTTGGCAGTA	562

rrs	16S-D	ACCGCCTACGCACCCTTTAC	16S-G	ACACTGGGACTGAGACACGG	277

Supplementary Table S2. List of representative organisms from Alpha-, Beta-, Gamma-, Delta-Proteobacteria. For each phylum, 20 organisms were selected.

Class	Order	Family	Genus	Name of organism
Alpha-Proteobacteria	Caulobacterales	Caulobacteraceae	*Brevundimonas*	*Brevundimonas subvibrioides* ATCC 15264
			Caulobacter	*Caulobacter crescentus* CB15
	Rhizobiales	Bartonellaceae	*Bartonella*	*Bartonella bacilliformis* KC583
		Beijerinckiaceae	*Methylocella*	*Methylocella silvestris* BL2
		Bradyrhizobiaceae	*Nitrobacter*	*Nitrobacter hamburgensis* X14
		Brucellaceae	*Brucella*	*Brucella abortus* bv. 1 9-941
		Rhizobiaceae	*Rhizobium*	*Agrobacterium fabrum* C58
	Rhodobacterales	Hyphomonadaceae	*Hirschia*	*Hirschia baltica* ATCC 49814
			Hyphomonas	*Hyphomonas neptunium* ATCC 15444
		Rhodobacteraceae	*Rhodobacter*	*Rhodobacter capsulatus* SB 1003
	Rhodospirillales	Acetobacteraceae	*Acidiphilium*	*Acidiphilium cryptum* JF-5
			Gluconacetobacter	*Gluconacetobacter diazotrophicus* Pal 5
			Gluconobacter	*Gluconobacter oxydan*s 621H
			Granulibacter	*Granulibacter bethesdensis* CGDNIH1
		Rhodospirillaceae	*Rhodospirillum*	*Rhodospirillum rubrum* ATCC 11170
	Rickettsiales	Anaplasmataceae	*Ehrlichia*	*Ehrlichia canis* Jake
	Sphingomonadales	Erythrobacteraceae	*Erythrobacter*	*Erythrobacter litoralis* HTCC2594
		Sphingomonadaceae	*Novosphingobium*	*Novosphingobium aromaticivorans* DSM 12444
			Sphingobium	*Sphingobium chlorophenolicum* L-1
			Sphingopyxis	*Sphingopyxis alaskensis* RB2256
Beta-Proteobacteria	Burkholderiales	Alcaligenaceae	*Bordetella*	*Bordetella pertussis* Tohama I
			Taylorella	*Taylorella equigenitalis* MCE9
		Burkholderiaceae	*Burkholderia*	*Burkholderia mallei* ATCC 23344
			Polynucleobacter	*Polynucleobacter necessarius* STIR1
			Ralstonia	*Ralstonia pickettii* 12D
		Comamonadaceae	*Acidovorax*	*Acidovorax citrulli* AAC00-1
			Polaromonas	*Polaromonas naphthalenivorans* CJ2
			Ramlibacter	*Ramlibacter tataouinensis* TTB310

			Rhodoferax	*Rhodoferax ferrireducens* T118
			Verminephrobacter	*Verminephrobacter eiseniae* EF01-2
		Oxalobacteraceae	*Herbaspirillum*	*Herbaspirillum seropedicae* SmR1
	Gallionellales	Gallionellaceae	*Gallionella*	*Gallionella capsiferriformans* ES-2
			Sideroxydans	*Sideroxydans lithotrophicus* ES-1
	Hydrogenophilales	Hydrogenophilaceae	*Thiobacillus*	*Thiobacillus denitrificans* ATCC 25259
	Methylophilales	Methylophilaceae	*Methylobacillus*	*Methylobacillus flagellatus* KT
	Neisseriales	Chromobacteriaceae	*Laribacter*	*Laribacter hongkongensis* HLHK9
	Nitrosomonadales	Nitrosomonadaceae	*Nitrosomonas*	*Nitrosomonas europaea* ATCC 19718
			Nitrosospira	*Nitrosospira multiformis* ATCC 25196
	Rhodocyclales	Rhodocyclaceae	*Aromatoleum*	*Aromatoleum aromaticum* EbN1
			Dechloromonas	*Dechloromonas aromatica* RCB
Delta-Proteobacteria	Bdellovibrionales	Bdellovibrionaceae	*Bdellovibrio*	*Bdellovibrio bacteriovorus* HD100
	Desulfarculales	Desulfarculaceae	*Desulfarculus*	*Desulfarculus baarsii* DSM 2075
	Desulfobacterales	Desulfobacteraceae	*Desulfatibacillum*	*Desulfatibacillum alkenivorans* AK-01
			Desulfobacterium	*Desulfobacterium autotrophicum* HRM2
			Desulfobacula	*Desulfobacula toluolica* Tol2
			Desulfococcus	*Desulfococcus oleovorans* Hxd3
		Desulfobulbaceae	*Desulfobulbus*	*Desulfobulbus propionicus* DSM 2032
			Desulfocapsa	*Desulfocapsa sulfexigens* DSM 10523
			Desulfotalea	*Desulfotalea psychrophila* LSv54
			Desulfurivibrio	*Desulfurivibrio alkaliphilus* AHT2
	Desulfovibrionales	Desulfohalobiaceae	*Desulfohalobium*	*Desulfohalobium retbaense* DSM 5692
		Desulfomicrobiaceae	*Desulfomicrobium*	*Desulfomicrobium baculatum* DSM 4028
		Desulfovibrionaceae	*Desulfovibrio*	*Desulfovibrio vulgaris* Hildenborough
			Lawsonia	*Lawsonia intracellularis* PHE/MN1-00
	Desulfuromonadal-es	Geobacteraceae	*Geobacter*	*Geobacter bemidjiensis* Bem
	Myxococcales	Cystobacterineae	*Cystobacteraceae*	*Stigmatella aurantiaca* DW4/3-1
		Cystobacterineae	*Myxococcaceae*	*Anaeromyxobacter dehalogenans* 2CP-C
	Syntrophobacteral-es	Syntrophaceae	*Desulfobacca*	*Desulfobacca acetoxidans* DSM 11109
			Syntrophus	*Syntrophus aciditrophicus* SB

		Syntrophobacteraceae	*Syntrophobacter*	*Syntrophobacter fumaroxidans* MPOB
Gamma-Proteobacteria	Aeromonadales	Aeromonadaceae	*Tolumonas*	*Tolumonas auensis* DSM 9187
	Alteromonadales	Pseudoalteromonadace-ae	*Pseudoalteromonas*	*Pseudoalteromonas haloplanktis* TAC125
		Psychromonadaceae	*Psychromonas*	*Psychromonas ingrahamii* 37
	Enterobacteriales	Enterobacteriaceae	*Edwardsiella*	*Edwardsiella ictaluri* 93-146
			Erwinia	*Erwinia amylovora* CFBP1430
			Escherichia	*Escherichia coli* K12 substr. MG1655
			Proteus	*Proteus mirabilis* HI4320
			Sodalis	*Sodalis glossinidius* str. Morsitans
	Oceanospirillales	Alcanivoracaceae	*Alcanivorax*	*Alcanivorax borkumensis* SK2
		Halomonadaceae	*Chromohalobacter*	*Chromohalobacter salexigens* DSM 3043
			Halomonas	*Halomonas elongata* DSM 2581
		Marinomonas		*Marinomonas posidonica I*VIA-Po-181
	Pasteurellales	Pasteurellaceae	*Basfia*	*Mannheimia succiniciproducens* MBEL55E
			Gallibacterium	*Gallibacterium anatis* UMN179
			Haemophilus	*Haemophilus ducreyi* 35000HP
			Histophilus	*Haemophilus somnus 2336*
	Pseudomonadales	Moraxellaceae	*Acinetobacter*	*Acinetobacter baumannii* ATCC 17978
			Psychrobacter	*Psychrobacter arcticus* 273-4
	Thiotrichales	Piscirickettsiaceae	*Thioalkalimicrobium*	*Thioalkalimicrobium cyclicum* ALM1
	Vibrionales	Vibrionaceae	*Vibrio*	*Vibrio anguillarum* 775

Supplementary Table S3. Comparative genomic features of the *Acidithiobacillus* species.

	***At. ferrivorans* CF27**		***At. ferrivorans* SS3**	***At. ferrooxidans* ATCC 23270^{T}**	***At. ferrooxidans* ATCC 53993**	***At. thiooxidans* ATCC 19377^{T}**	***At. caldus* SM-1***	***At. caldus* ATCC 51756^{T}***
	Chromosome	Plasmid						
**Genome size (bp)**	3,409,655	46,453	3,207,552	2,982,397	2,885,038	3,036,168	3,237,599	2,987,045
**Number of contigs**	5	1	1	1	1	165	5	4
**Predicted CDS**	3838	50	3785	3608	3225	3123	3571	2933
**G+C content (%)**	56.5	57.3	56.6	58.8	58.8	53.1	61.0	61.4
**Coding density (%)**	90.04	72.31	87.7	90.3	89.7	89.0	88.5	87.7
**Means CDS length (bp)**	819.12	674.46	823	790.8	844	877.7	845.5	913
**Maximal CDS length (bp)**	8568	3108	5352	7461	7461	5490	4416	7029
**tRNAs**	73 (20)	0(20)	47 (20)	80 (20)	46 (20)	43 (20)	47 (20)	49 (20)
**5S/16S/23S rRNA**	02/02/02	0	02/02/02	02/02/02	02/02/02	02/01/01	02/02/02	02/02/02
**Proteins assigned to COGs**	2482(64.7%)	33(66.0%)	2434(64.3%)	2341(64.9%)	2346(72.7%)	2364(75.7%)	2461(68.9%)	2172(74.1%)
**Proteins involved in**								
Replication Recombination and Repair (L)	260(6.8%)	5(10.0%)	252(6.6%)	223(6.2%)	258(8.0%)	221(7.1%)	385(10.8%)	297(10.1%)
Cell wall/membrane/envelope biogenesis (M)	203(5.2%)	5(10.0%)	209(5.5%)	216(6.0%)	203(6.3%)	232(7.4%)	211(5.9%)	190(6.5%)
Amino acid Transport and Metabolism (E)	204(5.3%)	2(4.0%)	195(5.1%)	198(5.5%)	200(6.2%)	195(6.2%)	191(5.3%)	180(6.1%)
Energy production and conversion (C)	212(5.5%)	1(2.0%)	198(5.2%)	191(5.3%)	197(6.1%)	184(5.9%)	192(5.4%)	183(6.2%)
Inorganic Ion Transport Metabolism (P)	201(5.2%)	2(4.0%)	177(4.7%)	185(5.1%)	196(6.1%)	162(5.2%)	174(4.9%)	136(4.6%)

*Data are generated from both chromosomes and plasmids. Data are from MicroScope except *At. caldus* ATCC 51756^{T}.

Supplemental Table S4. Predicted Genomic Islands (GI) within *At. ferrivorans* CF27 genome.

Island no	Island start	Island end	Length (kb)	GC content (%)	Locus	Strand	Product
1	177715	187669	10.0	55.9	AFERRI_v2_10165	-	putative N6-adeinine specific methyltransferase
					AFERRI_v2_10166	-	putative transcriptional regulator
					AFERRI_v2_10167	-	conserved protein of unknown function
					AFERRI_v2_10168	-	AAA ATPase central domain protein
					AFERRI_v2_10169	+	protein of unknown function
					AFERRI_v2_10170	+	protein of unknown function
					AFERRI_v2_10171	-	conserved protein of unknown function
					AFERRI_v2_10172	+	conserved protein of unknown function
					AFERRI_v2_10173	-	conserved protein of unknown function
					AFERRI_v2_10174	-	conserved membrane protein of unknown function
					AFERRI_v2_10175	-	protein of unknown function
					AFERRI_v2_10176	+	protein of unknown function
2	207094	224836	17.7	53.5	AFERRI_v2_10196	+	protein of unknown function
					AFERRI_v2_10197	+	protein of unknown function
					AFERRI_v2_10198	-	protein of unknown function
					AFERRI_v2_10199	-	putative mobilization protein, MobD
					AFERRI_v2_10200	-	protein of unknown function
					AFERRI_v2_10201	-	protein of unknown function
					AFERRI_v2_10202	-	protein of unknown function
					AFERRI_v2_10203	-	protein of unknown function
					AFERRI_v2_10204	+	protein of unknown function
					AFERRI_v2_10205	+	protein of unknown function
					AFERRI_v2_10206	+	protein of unknown function
					AFERRI_v2_10207	+	protein of unknown function
					AFERRI_v2_10208	+	putative TraI protein
					AFERRI_v2_10209	+	protein of unknown function
					AFERRI_v2_10210	+	membrane protein of unknown function
					AFERRI_v2_10211	+	protein of unknown function
					AFERRI_v2_10212	+	conserved protein of unknown function
					AFERRI_v2_10213	+	protein of unknown function
					AFERRI_v2_10214	+	prepilin
					AFERRI_v2_10215	-	membrane protein of unknown function
					AFERRI_v2_10216	-	tRNA(fMet)-specific endonuclease VapC
3	306302	312432	6.1	59.7	AFERRI_v2_10311	+	conserved protein of unknown function
					AFERRI_v2_10312	+	protein of unknown function
					AFERRI_v2_10313	-	conserved protein of unknown function

					AFERRI_v2_10314	-	putative cadmium-transporting ATPase
					AFERRI_v2_10315	-	transcriptional regulator, ArsR family
					AFERRI_v2_10316	-	putative Co/Zn/Cd cation efflux protein CzcD (fragment)
					AFERRI_v2_10317	-	conserved exported protein of unknown function
					AFERRI_v2_10318	-	extracellular solute-binding protein family 1
4	447438	452314	4.9	51.9	AFERRI_v2_10482	-	conserved protein of unknown function
					AFERRI_v2_10483	-	conserved protein of unknown function
					AFERRI_v2_10484	-	protein of unknown function
					AFERRI_v2_10485	-	conserved protein of unknown function
					AFERRI_v2_10486	-	conserved protein of unknown function
					AFERRI_v2_10487	-	conserved protein of unknown function
					AFERRI_v2_10488	-	protein of unknown function
5	457085	490536	33.5	54.5	AFERRI_v2_10495	+	conserved protein of unknown function
					AFERRI_v2_10496	+	type 4 prepilin-like proteins leader peptide-processing enzyme
					AFERRI_v2_10497	-	UvrD/REP helicase
					AFERRI_v2_10498	-	protein of unknown function
					AFERRI_v2_10499	+	protein of unknown function
					AFERRI_v2_10500	+	conserved protein of unknown function
					AFERRI_v2_10501	-	cell filamentation protein
					AFERRI_v2_10502	+	protein of unknown function
					AFERRI_v2_10503	-	conserved protein of unknown function
					AFERRI_v2_10504	-	conserved exported protein of unknown function
					AFERRI_v2_10505	-	protein of unknown function
					AFERRI_v2_10506	-	integration host factor subunit beta
					AFERRI_v2_10507	+	protein of unknown function
					AFERRI_v2_10508	-	conserved protein of unknown function
					AFERRI_v2_10509	-	conserved protein of unknown function
					AFERRI_v2_10510	+	conserved protein of unknown function
					AFERRI_v2_10511	-	conserved protein of unknown function
					AFERRI_v2_10512	-	protein of unknown function
					AFERRI_v2_10513	-	putative nicotinate phosphoribosyltransferase (modular protein)
					AFERRI_v2_10514	-	bifunctional NMN adenylyltransferase/Nudix hydrolase
					AFERRI_v2_10515	+	protein of unknown function
					AFERRI_v2_10516	-	protein of unknown function
					AFERRI_v2_10517	-	protein of unknown function
					AFERRI_v2_10518	-	protein of unknown function
					AFERRI_v2_10519	-	gap repair protein (fragment)
					AFERRI_v2_10520	-	ATP-dependent zinc metalloprotease FtsH (fragment)

					AFERRI_v2_10521	-	protein of unknown function
					AFERRI_v2_10522	-	protein of unknown function
					AFERRI_v2_10523	-	protein of unknown function
					AFERRI_v2_10524	-	5’ nucleotidase, deoxy, cytosolic type C
					AFERRI_v2_10525	-	conserved protein of unknown function
					AFERRI_v2_10526	-	conserved protein of unknown function
					AFERRI_v2_10527	-	DNA topoisomerase 4 subunit B
					AFERRI_v2_10530	-	conserved protein of unknown function
					AFERRI_v2_10531	-	conserved protein of unknown function
					AFERRI_v2_10532	-	phosphonopyruvate decarboxylase
					AFERRI_v2_10533	-	aspartate carbamoyltransferase
					AFERRI_v2_10534	-	putative phosphonoacetaldehyde dehydrogenase
					AFERRI_v2_10535	-	phosphoenolpyruvate phosphomutase (fragment)
					AFERRI_v2_10536	-	conserved protein of unknown function
					AFERRI_v2_10537	-	HNH endonuclease-like protein (fragment)
					AFERRI_v2_10538	+	protein of unknown function
					AFERRI_v2_10539	-	protein of unknown function
					AFERRI_v2_10540	-	type IV pilus assembly PilZ
6	501877	509724	7.8	56.3	AFERRI_v2_10553	-	conserved protein of unknown function
					AFERRI_v2_10554	-	DNA helicase (modular protein)
					AFERRI_v2_10555	+	protein of unknown function
					AFERRI_v2_10556	-	conserved protein of unknown function
					AFERRI_v2_10557	-	putative exodeoxyribonuclease X
					AFERRI_v2_10558	-	protein of unknown function
					AFERRI_v2_10559	-	protein of unknown function
					AFERRI_v2_10560	-	protein of unknown function
					AFERRI_v2_10561	-	conserved protein of unknown function
					AFERRI_v2_10562	-	DnaJ domain-containing protein
					AFERRI_v2_10563	-	conserved protein of unknown function
7	513505	517660	4.2	56.3	AFERRI_v2_10571	+	conserved protein of unknown function
					AFERRI_v2_10572	+	conserved protein of unknown function
					AFERRI_v2_10573	+	transglycosylase, putative (modular protein)
					AFERRI_v2_10574	+	conserved protein of unknown function
					AFERRI_v2_10575	+	conserved protein of unknown function
					AFERRI_v2_10576	+	protein of unknown function
					AFERRI_v2_10577	-	conserved protein of unknown function
					AFERRI_v2_10578	+	conserved membrane protein of unknown function
					AFERRI_v2_10579	-	HNH endonuclease domain protein (fragment)
8	529966	534305	4.3	55.2	AFERRI_v2_10601	+	protein of unknown function
					AFERRI_v2_10602	-	TraT complement resistance protein

					AFERRI_v2_10603	-	17 kDa surface antigen
					AFERRI_v2_10604	-	putative nucleoside 2-deoxyribosyltransferase family protein
					AFERRI_v2_10605	-	protein of unknown function
					AFERRI_v2_10606	-	protein of unknown function
					AFERRI_v2_10607	-	conserved protein of unknown function
					AFERRI_v2_10608	-	protein of unknown function
					AFERRI_v2_10609	-	ErfK/YbiS/YcfS/YnhG family protein
9	573762	588958	15.2	56.5	AFERRI_v2_10681	-	conserved protein of unknown function
					AFERRI_v2_10682	-	protein of unknown function
					AFERRI_v2_10683	-	protein of unknown function
					AFERRI_v2_10684	-	exported protein of unknown function
					AFERRI_v2_10685	-	DNA-binding protein HU
					AFERRI_v2_10686	-	protein of unknown function
					AFERRI_v2_10687	-	tRNA/rRNA methyltransferase
					AFERRI_v2_10688	-	protein of unknown function
					AFERRI_v2_10689	-	protein of unknown function
					AFERRI_v2_10690	-	D12 class N6 adenine-specific DNA methyltransferase (modular protein)
					AFERRI_v2_10691	-	conserved protein of unknown function
					AFERRI_v2_10692	-	conserved protein of unknown function
					AFERRI_v2_10693	+	protein of unknown function
					AFERRI_v2_10694	+	protein of unknown function
					AFERRI_v2_10695	-	CRISPR-associated protein Cas2
					AFERRI_v2_10696	-	conserved hypothetical protein
					AFERRI_v2_10697	-	conserved protein of unknown function
					AFERRI_v2_10698	-	conserved protein of unknown function
					AFERRI_v2_10699	-	conserved protein of unknown function
					AFERRI_v2_10700	-	protein of unknown function
10	602474	633841	31.4	54.1	AFERRI_v2_10720	-	putative Acyl carrier protein
					AFERRI_v2_10721	-	metallophosphoesterase (modular protein)
					AFERRI_v2_10722	-	conserved protein of unknown function
					AFERRI_v2_10723	+	protein of unknown function
					AFERRI_v2_10724	+	conserved protein of unknown function
					AFERRI_v2_10725	+	conserved protein of unknown function
					AFERRI_v2_10726	-	protein of unknown function
					AFERRI_v2_10727	+	conserved protein of unknown function
					AFERRI_v2_10728	+	conserved protein of unknown function
					AFERRI_v2_10729	+	putative plasmid-like protein
					AFERRI_v2_10730	+	conserved protein of unknown function
					AFERRI_v2_10731	+	conserved protein of unknown function
					AFERRI_v2_10732	+	protein of unknown function

					AFERRI_v2_10733	+	conserved protein of unknown function
					AFERRI_v2_10734	+	protein of unknown function
					AFERRI_v2_10735	+	protein of unknown function
					AFERRI_v2_10736	+	conserved protein of unknown function
					AFERRI_v2_10737	+	protein of unknown function
					AFERRI_v2_10738	-	protein of unknown function
					AFERRI_v2_10739	+	protein of unknown function
					AFERRI_v2_10740	+	protein of unknown function
					AFERRI_v2_10741	+	conserved protein of unknown function
					AFERRI_v2_10742	+	conserved protein of unknown function
					AFERRI_v2_10743	+	protein of unknown function
					AFERRI_v2_10744	+	protein of unknown function
					AFERRI_v2_10745	-	protein of unknown function
					AFERRI_v2_10746	+	conserved protein of unknown function
					AFERRI_v2_10747	+	conserved protein of unknown function
					AFERRI_v2_10748	+	protein of unknown function
					AFERRI_v2_10749	+	DNA ligase
					AFERRI_v2_10750	+	HNH endonuclease domain protein
					AFERRI_v2_10751	+	transcriptional regulator, XRE family (fragment)
					AFERRI_v2_10752	-	conserved protein of unknown function
					AFERRI_v2_10753	+	protein of unknown function
					AFERRI_v2_10754	-	single-stranded-DNA-specific exonuclease RecJ
					AFERRI_v2_10755	-	conserved membrane protein of unknown function
					AFERRI_v2_10756	-	membrane protein of unknown function
					AFERRI_v2_10757	-	phospholipid/glycerol acyltransferase
					AFERRI_v2_10758	-	conserved protein of unknown function
					AFERRI_v2_10759	-	protein of unknown function
					AFERRI_v2_10760	-	thiol:disulfide interchange protein DsbG, putative (modular protein)
					AFERRI_v2_10761	-	RNA polymerase sigma-70 factor family protein
					AFERRI_v2_10762	-	HNH endonuclease domain protein
					AFERRI_v2_10763	-	protein of unknown function
					AFERRI_v2_10764	-	protein of unknown function
					AFERRI_v2_10765	-	protein of unknown function
					AFERRI_v2_10766	-	putative fimbrial family protein
					AFERRI_v2_10767	-	conserved protein of unknown function
					AFERRI_v2_10768	-	conserved protein of unknown function
					AFERRI_v2_10769	-	membrane protein of unknown function
					AFERRI_v2_10770	-	type IV pilus assembly protein TapC
11	636939	645667	8.7	54.7	AFERRI_v2_10773	-	protein of unknown function
					AFERRI_v2_10774	-	conserved membrane protein of unknown

							function
					AFERRI_v2_10775	-	conserved protein of unknown function
					AFERRI_v2_10776	-	DNA helicase II (modular protein)
					AFERRI_v2_10777	-	conserved protein of unknown function
					AFERRI_v2_10778	+	protein of unknown function
					AFERRI_v2_10779	-	protein of unknown function
					AFERRI_v2_10780	-	conserved protein of unknown function
					AFERRI_v2_10781	-	conserved membrane protein of unknown function
					AFERRI_v2_10782	+	protein of unknown function
					AFERRI_v2_10783	-	conserved protein of unknown function
					AFERRI_v2_10784	-	conserved membrane protein of unknown function
					AFERRI_v2_10785	-	putative DNA replication protein DnaC
					AFERRI_v2_10786	-	replication protein 15
12	646860	658511	11.7	56.5	AFERRI_v2_10788	-	conserved protein of unknown function
					AFERRI_v2_10789	-	conserved protein of unknown function
					AFERRI_v2_10790	-	protein of unknown function
					AFERRI_v2_10791	-	site-specific DNA methylase
					AFERRI_v2_10792	-	conserved protein of unknown function
					AFERRI_v2_10793	-	conserved protein of unknown function
					AFERRI_v2_10794	-	protein of unknown function
					AFERRI_v2_10795	-	conserved protein of unknown function
					AFERRI_v2_10796	-	conserved protein of unknown function
					AFERRI_v2_10797	-	conserved exported protein of unknown function
					AFERRI_v2_10798	-	conserved protein of unknown function
					AFERRI_v2_10799	-	StbA family protein
					AFERRI_v2_10800	+	relaxase (modular protein)
					AFERRI_v2_10801	+	HNH endonuclease-like protein
13	665702	674225	8.5	53.4	AFERRI_v2_10808	+	HNH endonuclease domain protein
					AFERRI_v2_10809	+	conserved membrane protein of unknown function
					AFERRI_v2_10810	+	type IV secretory pathway VirB3 family protein
					AFERRI_v2_10811	+	type IV secretory pathway VirB4 components-like protein
					AFERRI_v2_10812	+	conserved exported protein of unknown function
					AFERRI_v2_10813	+	conserved membrane protein of unknown function
					AFERRI_v2_10814	+	conserved protein of unknown function
					AFERRI_v2_10815	-	exported protein of unknown function
					AFERRI_v2_10816	-	membrane protein of unknown function
14	679862	708612	28.8	56.0	AFERRI_v2_10824	-	AAA ATPase (modular protein)
					AFERRI_v2_10825	+	conserved protein of unknown function

					AFERRI_v2_10826	+	protein of unknown function
					AFERRI_v2_10827	-	protein of unknown function
					AFERRI_v2_10828	-	conserved exported protein of unknown function
					AFERRI_v2_10829	-	exported protein of unknown function
					AFERRI_v2_10830	+	protein of unknown function
					AFERRI_v2_10831	-	conserved protein of unknown function
					AFERRI_v2_10832	+	type IV secretory pathway TrbF protein-like protein
					AFERRI_v2_10833	+	conjugal transfer protein TrbG/VirB9/CagX
					AFERRI_v2_10834	+	conjugation TrbI family protein
					AFERRI_v2_10835	+	protein of unknown function
					AFERRI_v2_10836	+	membrane protein of unknown function
					AFERRI_v2_10837	-	protein of unknown function
					AFERRI_v2_10838	+	conserved protein of unknown function
					AFERRI_v2_10839	+	conserved protein of unknown function
					AFERRI_v2_10840	+	exported protein of unknown function
					AFERRI_v2_10841	+	lytic transglycosylase, catalytic (modular protein)
					AFERRI_v2_10842	+	membrane protein of unknown function
					AFERRI_v2_10843	+	conserved protein of unknown function
					AFERRI_v2_10844	+	protein of unknown function
					AFERRI_v2_10845	+	EAL domain protein
					AFERRI_v2_10846	+	protein of unknown function
					AFERRI_v2_10847	+	PilN
					AFERRI_v2_10848	+	exported protein of unknown function
					AFERRI_v2_10849	+	protein of unknown function
					AFERRI_v2_10850	+	protein of unknown function
					AFERRI_v2_10851	+	putative type IV secretion apparatus, ATPase component (PilQ homolog)
					AFERRI_v2_10852	+	putative Type II secretion system protein
					AFERRI_v2_10853	+	protein of unknown function
					AFERRI_v2_10854	+	putative Type IV prepropilin
					AFERRI_v2_10855	+	putative PilU
					AFERRI_v2_10856	+	exported protein of unknown function
					AFERRI_v2_10857	+	prepilin
					AFERRI_v2_10858	-	membrane protein of unknown function
					AFERRI_v2_10859	-	protein of unknown function
					AFERRI_v2_10860	-	conserved exported protein of unknown function
					AFERRI_v2_10861	-	protein of unknown function
					AFERRI_v2_10862	-	conserved protein of unknown function
15	719674	750545	30.9	53.4	AFERRI_v2_10875	-	diguanylate cyclase/phosphodiesterase with PAS/PAC sensor(S)
					AFERRI_v2_10876	+	conserved protein of unknown function

					AFERRI_v2_10877	+	protein of unknown function
					AFERRI_v2_10878	+	protein of unknown function
					AFERRI_v2_10879	+	conserved protein of unknown function
					AFERRI_v2_10880	+	UvrD/REP helicase (modular protein)
					AFERRI_v2_10881	+	conjugal transfer protein TraB (modular protein)
					AFERRI_v2_10882	+	conserved protein of unknown function
					AFERRI_v2_10883	-	conserved protein of unknown function
					AFERRI_v2_10884	+	single-stranded DNA-binding protein
					AFERRI_v2_10885	+	conserved protein of unknown function
					AFERRI_v2_10886	+	conserved protein of unknown function
					AFERRI_v2_10887	+	DNA repair exonuclease-like protein
					AFERRI_v2_10888	+	conserved protein of unknown function
					AFERRI_v2_10889	+	conserved protein of unknown function
					AFERRI_v2_10890	+	putative ATPase involved in DNA repair
					AFERRI_v2_10891	+	conserved protein of unknown function
					AFERRI_v2_10892	+	conserved protein of unknown function
					AFERRI_v2_10893	-	protein of unknown function
					AFERRI_v2_10894	+	conserved protein of unknown function
					AFERRI_v2_10895	+	conserved exported protein of unknown function
					AFERRI_v2_10896	+	conserved protein of unknown function
					AFERRI_v2_10897	+	conserved protein of unknown function
					AFERRI_v2_10898	+	conserved protein of unknown function
					AFERRI_v2_10899	+	conserved protein of unknown function
					AFERRI_v2_10900	+	protein of unknown function
					AFERRI_v2_10901	+	conserved exported protein of unknown function
					AFERRI_v2_10902	+	conserved protein of unknown function
					AFERRI_v2_10903	+	conserved protein of unknown function
					AFERRI_v2_10904	+	conserved protein of unknown function
					AFERRI_v2_10905	+	conserved protein of unknown function
					AFERRI_v2_10906	+	conserved protein of unknown function
					AFERRI_v2_10907	+	ribonucleoside-diphosphate reductase subunit beta
					AFERRI_v2_10908	+	protein of unknown function
					AFERRI_v2_10909	+	conserved protein of unknown function
					AFERRI_v2_10910	-	protein of unknown function
					AFERRI_v2_10911	+	conserved protein of unknown function
					AFERRI_v2_10912	+	conserved protein of unknown function
					AFERRI_v2_10913	+	conserved protein of unknown function
					AFERRI_v2_10914	+	protein of unknown function
					AFERRI_v2_10915	+	PRTRC system protein E
					AFERRI_v2_10916	+	conserved protein of unknown function

					AFERRI_v2_10917	+	protein of unknown function
					AFERRI_v2_10918	+	conserved protein of unknown function
16	759205	772269	13.1	53.2	AFERRI_v2_10927	+	RNA-directed DNA polymerase (Reverse transcriptase)
					AFERRI_v2_10928	+	Yea (fragment)
					AFERRI_v2_10929	+	protein of unknown function
					AFERRI_v2_10930	+	ribonucleoside-diphosphate reductase (modular protein)
					AFERRI_v2_10931	+	protein of unknown function
					AFERRI_v2_10932	+	protein of unknown function
					AFERRI_v2_10933	+	conserved protein of unknown function
					AFERRI_v2_10934	+	protein of unknown function
					AFERRI_v2_10935	-	protein of unknown function
					AFERRI_v2_10936	+	conserved protein of unknown function
					AFERRI_v2_10937	-	protein of unknown function
					AFERRI_v2_10938	+	protein of unknown function
					AFERRI_v2_10939	+	conserved protein of unknown function
					AFERRI_v2_10940	-	protein of unknown function
					AFERRI_v2_10941	+	ParB-like partition protein
					AFERRI_v2_10942	+	protein of unknown function
					AFERRI_v2_10943	+	protein of unknown function
					AFERRI_v2_10944	+	phage P4 alpha zinc-binding domain-containing protein (modular protein)
					AFERRI_v2_10945	+	DNA binding protein, excisionase family
					AFERRI_v2_10946	+	prophage DLP12 integrase
17	840276	844839	4.6	45.4	AFERRI_v2_11013	-	cytochrome *c* oxidase caa_3-type, assembly factor CtaG-related protein
					AFERRI_v2_11014	-	putative lipoprotein
					AFERRI_v2_11015	+	protein of unknown function
					AFERRI_v2_11016	-	conserved protein of unknown function
					AFERRI_v2_11017	-	protein of unknown function
					AFERRI_v2_11018	+	DsbG domain protein
					AFERRI_v2_11019	-	conserved protein of unknown function
18	1073931	1082115	8.2	53.3	AFERRI_v2_11244	-	conserved protein of unknown function
					AFERRI_v2_11245	-	conserved protein of unknown function
					AFERRI_v2_11246	-	conserved protein of unknown function
					AFERRI_v2_11247	-	conserved protein of unknown function
					AFERRI_v2_11248	-	protein of unknown function
					AFERRI_v2_11249	-	protein of unknown function
					AFERRI_v2_11250	-	protein of unknown function
					AFERRI_v2_11251	-	DNA methylase N-4/N-6 domain protein
					AFERRI_v2_11252	-	conserved protein of unknown function
					AFERRI_v2_11253	-	type III restriction protein res subunit

19	1159095	1163968	4.9	54.7	AFERRI_v2_20090	+	phage integrase
					AFERRI_v2_20091	+	conserved protein of unknown function
					AFERRI_v2_20092	+	transcriptional regulator, XRE family
					AFERRI_v2_20093	+	protein of unknown function
					AFERRI_v2_20094	+	protein of unknown function
					AFERRI_v2_20095	-	protein of unknown function
20	1191531	1205578	14.0	54.6	AFERRI_v2_20132	+	NADH:ubiquinone oxidoreductase, chain C,D
					AFERRI_v2_20133	+	NADH:ubiquinone oxidoreductase, chain E
					AFERRI_v2_20134	+	NADH:ubiquinone oxidoreductase, chain F
					AFERRI_v2_20135	+	NADH:ubiquinone oxidoreductase, chain G
					AFERRI_v2_20136	+	NADH:ubiquinone oxidoreductase, membrane subunit H
					AFERRI_v2_20137	+	NADH dehydrogenase I chain I, 2Fe-2S ferredoxin-related
					AFERRI_v2_20138	+	NADH:ubiquinone oxidoreductase, membrane subunit J
					AFERRI_v2_20139	+	NADH:ubiquinone oxidoreductase, membrane subunit K
					AFERRI_v2_20140	+	NADH:ubiquinone oxidoreductase, membrane subunit L
					AFERRI_v2_20141	+	NADH:ubiquinone oxidoreductase, membrane subunit M
					AFERRI_v2_20142	+	NADH:ubiquinone oxidoreductase, membrane subunit N
21	1279556	1284218	4.7	56.7	AFERRI_v2_20217	+	conserved protein of unknown function
					AFERRI_v2_20218	+	phage-related DNA-binding protein
					AFERRI_v2_20219	+	conserved protein of unknown function
					AFERRI_v2_20220	+	conserved protein of unknown function
					AFERRI_v2_20221	-	transposase (fragment)
					AFERRI_v2_20222	-	protein of unknown function
					AFERRI_v2_20223	-	phage integrase family protein
					AFERRI_v2_20224	-	protein of unknown function
22	1325229	1331534	6.3	56.6	AFERRI_v2_20272	+	integrase family protein
					AFERRI_v2_20273	+	protein of unknown function
					AFERRI_v2_20274	+	protein of unknown function
					AFERRI_v2_20275	+	protein of unknown function
					AFERRI_v2_20276	+	conserved protein of unknown function
					AFERRI_v2_20277	+	protein of unknown function
					AFERRI_v2_20278	+	protein of unknown function
					AFERRI_v2_20279	+	protein of unknown function
					AFERRI_v2_20280	+	protein of unknown function
					AFERRI_v2_20281	-	conserved exported protein of unknown function
23	1340506	1352467	12.0	57.2	AFERRI_v2_20291	+	protein of unknown function
					AFERRI_v2_20292	-	type II site-specific deoxyribonuclease

					AFERRI_v2_20293	-	DNA cytosine methylase
					AFERRI_v2_20294	+	protein of unknown function
					AFERRI_v2_20295	+	protein of unknown function
					AFERRI_v2_20296	+	conserved protein of unknown function
					AFERRI_v2_20297	+	protein of unknown function
					AFERRI_v2_20298	+	conserved membrane protein of unknown function
					AFERRI_v2_20299	+	protein of unknown function
					AFERRI_v2_20300	+	protein of unknown function
					AFERRI_v2_20301	-	protein of unknown function
					AFERRI_v2_20302	-	protein of unknown function
					AFERRI_v2_20303	-	protein of unknown function
					AFERRI_v2_20304	-	resolvase domain protein (modular protein)
					AFERRI_v2_20305	+	putative bifunctional enzyme and transcriptional regulator
24	1479450	1485081	5.6	50.6	AFERRI_v2_20448	+	conserved protein of unknown function
					AFERRI_v2_20449	+	protein of unknown function
					AFERRI_v2_20450	+	DNA polymerase beta domain protein region
					AFERRI_v2_20451	+	conserved protein of unknown function
					AFERRI_v2_20452	+	transposase (fragment)
					AFERRI_v2_20453	-	conserved protein of unknown function
					AFERRI_v2_20454	-	conserved protein of unknown function
					AFERRI_v2_20455	-	conserved protein of unknown function
					AFERRI_v2_20456	-	glutathione-dependent formaldehyde-activating GFA (fragment)
					AFERRI_v2_20457	-	protein of unknown function
					AFERRI_v2_20458	-	exported protein of unknown function
					AFERRI_v2_20459	+	putative lipoprotein
25	1488917	1493246	4.3	42.8	AFERRI_v2_20469	+	pyrimidine dimer DNA glycosylase
					AFERRI_v2_20470	+	membrane protein of unknown function
					AFERRI_v2_20471	-	protein of unknown function
					AFERRI_v2_20472	-	membrane protein of unknown function
					AFERRI_v2_20473	-	conserved protein of unknown function
					AFERRI_v2_20474	-	protein of unknown function
					AFERRI_v2_20475	-	conserved protein of unknown function
26	1549714	1554467	4.8	60.1	AFERRI_v2_20548	-	transposase
					AFERRI_v2_20549	-	protein of unknown function
					AFERRI_v2_20550	-	transposase (fragment)
					AFERRI_v2_20551	+	putative site-specific recombinase; Qin prophage
					AFERRI_v2_20552	+	conserved protein of unknown function
					AFERRI_v2_20553	-	protein of unknown function
					AFERRI_v2_20554	+	conserved protein of unknown function

27	1690829	1695715	4.9	60.8	AFERRI_v2_20710	-	restriction modification system DNA specificity domain protein
					AFERRI_v2_20711	-	conserved protein of unknown function
					AFERRI_v2_20712	-	ATP-dependent DNA helicase
					AFERRI_v2_20713	-	type I restriction-modification system, R subunit
					AFERRI_v2_20714	+	protein of unknown function
28	1819579	1824099	4.5	50.3	AFERRI_v2_20840	-	protein of unknown function
					AFERRI_v2_20841	-	conserved protein of unknown function
					AFERRI_v2_20842	-	conserved protein of unknown function
					AFERRI_v2_20843	+	conserved protein of unknown function
					AFERRI_v2_20844	-	conserved exported protein of unknown function
29	1838123	1842392	4.3	59.5	AFERRI_v2_20857	+	transketolase 1, thiamin-binding
					AFERRI_v2_20858	+	D-erythrose 4-phosphate dehydrogenase
					AFERRI_v2_20859	+	phosphoglycerate kinase
					AFERRI_v2_20860	+	pyruvate kinase II
30	1965288	1970609	5.3	47.7	AFERRI_v2_20992	+	conserved protein of unknown function
					AFERRI_v2_20993	+	conserved protein of unknown function
					AFERRI_v2_20994	+	anti-sigma factor, ChrR (fragment)
					AFERRI_v2_20995	+	ribosyldihydronicotinamide dehydrogenase (Quinone)
					AFERRI_v2_20996	+	putative tautomerase K2
					AFERRI_v2_20997	+	conserved membrane protein of unknown function
					AFERRI_v2_20998	+	GCN5-like N-acetyltransferaser
					AFERRI_v2_20999	+	quinone oxidoreductase
					AFERRI_v2_21000	+	conserved protein of unknown function
31	1983847	1990712	6.9	61.5	AFERRI_v2_21016	+	protein of unknown function
					AFERRI_v2_21017	-	toxin of the YafQ-DinJ toxin-antitoxin system
					AFERRI_v2_21018	-	antitoxin of YafQ-DinJ toxin-antitoxin system
					AFERRI_v2_21019	+	conserved protein of unknown function
					AFERRI_v2_21020	+	exported protein of unknown function
					AFERRI_v2_21021	+	conserved protein of unknown function
					AFERRI_v2_21022	+	protein of unknown function
					AFERRI_v2_21023	+	integrase catalytic region (fragment)
					AFERRI_v2_21024	+	protein of unknown function
					AFERRI_v2_21025	+	prevent-host-death family protein
					AFERRI_v2_21026	+	conserved protein of unknown function
					AFERRI_v2_21027	+	protein of unknown function
					AFERRI_v2_21028	+	protein of unknown function
					AFERRI_v2_21029	+	protein of unknown function
32	2033189	2040096	6.9	48.4	AFERRI_v2_30014	-	protein of unknown function
					AFERRI_v2_30015	-	conserved protein of unknown function

					AFERRI_v2_30016	-	protein of unknown function
					AFERRI_v2_30017	-	RNA-directed DNA polymerase (reverse transcriptase) (fragment)
					AFERRI_v2_30018	+	RNA-directed DNA polymerase (reverse transcriptase) (fragment)
					AFERRI_v2_30019	+	regulatory protein TetR
					AFERRI_v2_30020	+	O-methyltransferase, family 2
					AFERRI_v2_30021	-	protein of unknown function
					AFERRI_v2_30022	-	GCN5-related N-acetyltransferase
					AFERRI_v2_30023	+	protein of unknown function
33	2099824	2105647	5.8	49.2	AFERRI_v2_30077	-	conserved protein of unknown function
					AFERRI_v2_30078	-	conserved protein of unknown function
					AFERRI_v2_30079	-	conserved protein of unknown function
					AFERRI_v2_30080	-	protein of unknown function
					AFERRI_v2_30081	-	protein of unknown function
					AFERRI_v2_30082	+	protein of unknown function
					AFERRI_v2_30083	-	conserved protein of unknown function
					AFERRI_v2_30084	+	protein of unknown function
					AFERRI_v2_30085	-	exported protein of unknown function
					AFERRI_v2_30086	-	conserved protein of unknown function
					AFERRI_v2_30087	-	protein of unknown function
34	2149077	2154223	5.1	59.2	AFERRI_v2_30128	+	high potential iron sulfur protein Hip
					AFERRI_v2_30129	+	conserved exported protein of unknown function
					AFERRI_v2_30130	+	protein of unknown function
					AFERRI_v2_30131	+	protein of unknown function
					AFERRI_v2_30132	+	conserved protein of unknown function
					AFERRI_v2_30133	+	hydroxymethylpyrimidine/phosphomethylpyrimidine kinase
					AFERRI_v2_30134	+	thiamine-phosphate synthase
					AFERRI_v2_30135	+	glutamate-1-semialdehyde aminotransferase (aminomutase)
					AFERRI_v2_30136	+	histidine triad (HIT) protein
					AFERRI_v2_30137	+	putative ComEA-like protein
35	2164758	2179516	14.8	52.9	AFERRI_v2_30152	+	protein of unknown function
					AFERRI_v2_30153	-	conserved protein of unknown function
					AFERRI_v2_30154	-	mannose-1-phosphate guanyltransferase
					AFERRI_v2_30155	-	GDP-D-mannose dehydratase, NAD(P)-binding
					AFERRI_v2_30156	-	GDP-6-deoxy-D-mannose reductase
					AFERRI_v2_30157	-	conserved protein of unknown function
					AFERRI_v2_30158	-	conserved protein of unknown function
					AFERRI_v2_30159	-	prevent-host-death family protein
					AFERRI_v2_30160	+	ABC-2 type transporter
					AFERRI_v2_30161	-	protein of unknown function

					AFERRI_v2_30162	+	O-antigen export system ATP-binding protein RfbB
					AFERRI_v2_30163	+	conserved protein of unknown function
					AFERRI_v2_30164	+	UbiA prenyltransferase
					AFERRI_v2_30165	+	protein of unknown function
					AFERRI_v2_30166	+	FAD linked oxidase domain protein
					AFERRI_v2_30167	+	short-chain dehydrogenase/reductase SDR
					AFERRI_v2_30168	+	membrane protein of unknown function
					AFERRI_v2_30169	+	conserved membrane protein of unknown function
					AFERRI_v2_30170	-	protein of unknown function
					AFERRI_v2_30171	-	putative insertion sequence ATP-binding protein Y4pL (fragment)
					AFERRI_v2_30172	+	conserved protein of unknown function
36	2228967	2234623	5.7	62.4	AFERRI_v2_30229	+	putative hydrogenase-4 component F homolog
					AFERRI_v2_30230	+	putative NADH dehydrogenase subunit E
					AFERRI_v2_30231	+	NADH ubiquinone oxidoreductase, 20 kDa subunit
					AFERRI_v2_30232	+	conserved membrane protein of unknown function
					AFERRI_v2_30233	+	conserved protein of unknown function
37	2243262	2251371	8.1	48.7	AFERRI_v2_30246	+	conserved protein of unknown function
					AFERRI_v2_30247	-	conserved protein of unknown function
					AFERRI_v2_30248	-	protein of unknown function
					AFERRI_v2_30249	-	protein of unknown function
					AFERRI_v2_30250	+	putative metallophosphoesterase
					AFERRI_v2_30251	+	conserved protein of unknown function
					AFERRI_v2_30252	+	protein of unknown function
					AFERRI_v2_30253	-	transposase (fragment)
38	2272111	2278166	6.1	48.1	AFERRI_v2_30285	+	protein of unknown function
					AFERRI_v2_30286	-	PRC-barrel domain protein
					AFERRI_v2_30287	-	CsbD family protein
					AFERRI_v2_30288	-	conserved exported protein of unknown function
					AFERRI_v2_30289	+	di-trans-poly-cis-decaprenylcistransferase
					AFERRI_v2_30290	-	conserved protein of unknown function
					AFERRI_v2_30291	+	conserved protein of unknown function
					AFERRI_v2_30292	+	major intrinsic protein
					AFERRI_v2_30293	-	conserved membrane protein of unknown function
39	2418627	2428928	10.3	51.3	AFERRI_v2_40072	+	integrase family protein
					AFERRI_v2_40073	+	protein of unknown function
					AFERRI_v2_40074	+	membrane protein of unknown function
					AFERRI_v2_40075	-	protein of unknown function
					AFERRI_v2_40076	-	putative helicase

					AFERRI_v2_40077	-	protein of unknown function
					AFERRI_v2_40078	-	VapC ribonuclease Y4jK
					AFERRI_v2_40079	-	putative plasmid stability protein
					AFERRI_v2_40080	-	conserved protein of unknown function
40	2436930	2447475	10.5	59.3	AFERRI_v2_40096	-	conserved protein of unknown function
					AFERRI_v2_40097	+	DNA topoisomerase type IA central domain protein
					AFERRI_v2_40098	+	conserved protein of unknown function
					AFERRI_v2_40099	+	single-stranded DNA-binding protein
					AFERRI_v2_40100	+	putative lipoprotein
					AFERRI_v2_40101	+	conserved protein of unknown function
					AFERRI_v2_40102	+	conserved protein of unknown function
					AFERRI_v2_40103	+	conserved protein of unknown function
					AFERRI_v2_40104	+	conserved protein of unknown function
					AFERRI_v2_40105	+	metal-dependent phosphohydrolase HD region
					AFERRI_v2_40106	+	conserved protein of unknown function
41	2735959	2740427	4.5	64.1	AFERRI_v2_50283	+	prevent-host-death family protein
					AFERRI_v2_50284	+	conserved protein of unknown function
					AFERRI_v2_50285	+	protein of unknown function
					AFERRI_v2_50286	+	conserved protein of unknown function
42	2820150	2826028	5.9	47.6	AFERRI_v2_50371	-	conserved protein of unknown function
					AFERRI_v2_50372	-	conserved protein of unknown function
					AFERRI_v2_50373	-	cytochrome c_1
					AFERRI_v2_50374	-	cytochrome *b*
					AFERRI_v2_50375	-	ubiquinol-cytochrome *c* reductase iron-sulfur subunit
					AFERRI_v2_50376	-	putative oxoacyl-(acyl carrier protein) reductase
					AFERRI_v2_50377	-	cytochrome *c* class I
					AFERRI_v2_50378	-	conserved protein of unknown function
43	2838903	2843176	4.3	56.0	AFERRI_v2_50390	+	putative type IIS restriction /modification enzyme, N-terminal half (fragment)
					AFERRI_v2_50391	+	SMC domain protein
					AFERRI_v2_50392	+	conserved protein of unknown function
44	2882842	2887373	4.5	60.2	AFERRI_v2_50431	+	FAD-dependent pyridine nucleotide-disulphide oxidoreductase
					AFERRI_v2_50432	+	putative oxidoreductase subunit
					AFERRI_v2_50433	+	D-hydantoinase/dihydropyrimidinase
					AFERRI_v2_50434	+	NCS1 nucleoside transporter
45	2889553	2894343	4.8	51.6	AFERRI_v2_50436	+	cytidine deaminase
					AFERRI_v2_50437	+	deoxyribose-phosphate aldolase protein
					AFERRI_v2_50438	+	uridine phosphorylase
					AFERRI_v2_50439	+	phosphomannomutase/phosphoglucomutase

					AFERRI_v2_50440	+	conserved protein of unknown function
					AFERRI_v2_50441	+	TonB-dependent receptor
46	2989606	3003885	14.3	46.7	AFERRI_v2_50538	+	protein of unknown function
					AFERRI_v2_50539	+	protein of unknown function
					AFERRI_v2_50540	+	conserved protein of unknown function
					AFERRI_v2_50541	+	conserved hypothetical protein
					AFERRI_v2_50542	+	conserved protein of unknown function
					AFERRI_v2_50543	+	3-ketoacyl-CoA reductase
					AFERRI_v2_50544	-	type-2 restriction enzyme PvuII
					AFERRI_v2_50545	-	subunit S of type I restriction-modification system
					AFERRI_v2_50546	+	modification methylase PvuII
					AFERRI_v2_50547	+	protein of unknown function
					AFERRI_v2_50548	+	NUDIX hydrolase
					AFERRI_v2_50549	-	protein of unknown function
					AFERRI_v2_50550	+	conserved protein of unknown function
					AFERRI_v2_50551	+	putative low-complexity protein
					AFERRI_v2_50552	+	nucleotidyltransferase family protein
					AFERRI_v2_50553	+	conserved protein of unknown function
					AFERRI_v2_50554	+	antitoxin YefM
					AFERRI_v2_50555	+	addiction module toxin, Txe/YoeB family
					AFERRI_v2_50556	+	conserved protein of unknown function
					AFERRI_v2_50557	+	conserved protein of unknown function
					AFERRI_v2_50558	+	protein of unknown function
					AFERRI_v2_50559	+	conserved protein of unknown function
					AFERRI_v2_50560	+	protein of unknown function
47	3092097	3100041	7.9	52.1	AFERRI_v2_50659	+	conserved exported protein of unknown function
					AFERRI_v2_50660	+	metallo-beta-lactamase family protein
					AFERRI_v2_50661	+	protein of unknown function
					AFERRI_v2_50662	+	cytochrome *c* oxidase subunit II
					AFERRI_v2_50663	+	cytochrome *o* ubiquinol oxidase subunit I
					AFERRI_v2_50664	+	cytochrome *c* oxidase subunit III
					AFERRI_v2_50665	+	cytochrome *o* ubiquinol oxidase, subunit IV
					AFERRI_v2_50666	+	conserved protein of unknown function
					AFERRI_v2_50667	+	transposase
					AFERRI_v2_50668	+	protein of unknown function
48	3120152	3133945	13.8	54.2	AFERRI_v2_50691	+	putative flagellar basal-body P-ring formation protein FlgA
					AFERRI_v2_50692	+	conserved protein of unknown function
					AFERRI_v2_50693	+	protein of unknown function
					AFERRI_v2_50694	-	putative RNA polymerase sigma factor 28 for flagellar operon, FliA

					AFERRI_v2_50695	-	putative flagellar biosynthesis protein FliQ
					AFERRI_v2_50696	-	flagellar biosynthesis protein
					AFERRI_v2_50697	-	flagellar motor switch protein FliN
					AFERRI_v2_50698	-	putative flagellar motor switch protein FliM
					AFERRI_v2_50699	-	protein of unknown function
					AFERRI_v2_50700	-	putative flagellar motor switch protein FliG
					AFERRI_v2_50701	-	putative flagellar M-ring protein FliF
					AFERRI_v2_50702	-	putative flagellar basal-body component FliE
					AFERRI_v2_50703	-	putative flagellin-specific chaperone FliS
					AFERRI_v2_50704	-	putative flagellar cap protein FliD
					AFERRI_v2_50705	-	putative flagellin protein FliC
					AFERRI_v2_50706	-	putative glycosyltransferase
49	3233450	3242046	8.6	56.0	AFERRI_v2_50812	-	protein of unknown function
					AFERRI_v2_50813	+	protein of unknown function
					AFERRI_v2_50814	+	protein of unknown function
					AFERRI_v2_50815	+	membrane protein of unknown function
					AFERRI_v2_50816	-	protein of unknown function
					AFERRI_v2_50817	-	protein of unknown function
					AFERRI_v2_50818	-	protein of unknown function
					AFERRI_v2_50819	-	predicted transcriptional regulator (fragment)
					AFERRI_v2_50820	+	protein of unknown function
					AFERRI_v2_50821	-	integrase family protein
50	3255990	3260578	4.6	57.2	AFERRI_v2_50839	+	glucose-1-phosphate cytidylyltransferase
					AFERRI_v2_50840	+	CDP-glucose 4,6-dehydratase
					AFERRI_v2_50841	+	dTDP-4-deoxyrhamnose-3,5-epimerase
					AFERRI_v2_50842	+	putative homoserine dehydrogenase
					AFERRI_v2_50843	-	acetyltransferase (modular protein)
51	3266550	3278744	12.2	55.9	AFERRI_v2_50849	-	GDP-6-deoxy-D-mannose reductase
					AFERRI_v2_50850	-	bifunctional GDP-fucose synthetase
					AFERRI_v2_50851	-	putative glycosyl transferase family 1, WcaI
					AFERRI_v2_50852	-	putative glycose-acyl transferase, WcaF
					AFERRI_v2_50853	-	putative glycosyl transferase, family 1
					AFERRI_v2_50854	-	transposase
					AFERRI_v2_50855	-	putative glycosyl transferase, family 1
					AFERRI_v2_50856	-	UDP-sulfoquinovose synthase
					AFERRI_v2_50857	-	protein of unknown function
					AFERRI_v2_50858	-	putative glycosyl transferase group 1
					AFERRI_v2_50859	-	putative glycosyl transferase group 1
					AFERRI_v2_50860	-	putative membrane protein
					AFERRI_v2_50861	-	putative glycosyl transferase group 1

					AFERRI_v2_50862	-	conserved protein of unknown function

Supplementary Table S5. Genes involved in cold adaptation, heavy metal resistance, motility and chemotaxis in *At. ferrivorans* CF27.

	**Gene**	**Gene ID**	**Protein function**
Cold adaptation	*treS*	AFERRI_v2_50367	Trehalose synthase
	glgX	AFERRI_v2_50368	Glycogen debranching enzyme
	treZ	AFERRI_v2_50369	Malto-oligosyltrehalose trehalohydrolase
	treY	AFERRI_v2_50370	Malto-oligosyltrehalose synthase TreY
	spsA	AFERRI_v2_10372	Sucrose-phosphate synthase
	susA	AFERRI_v2_10373	Sucrose synthase
	cspE	AFERRI_v2_20964, AFERRI_v2_50018, AFERRI_v2_50606	Cold shock protein
		AFERRI_v2_50281, AFERRI_v2_50465	DEAD/DEAH box helicase
	tig	AFERRI_v2_10133	Trigger factor (chaperone)
	surA	AFERRI_v2_50037	Membrane associated chaperone survival protein
	gtrB	AFERRI_v2_11131	Bactoprenol glucosyl transferase
	desA	AFERRI_v2_10410	Fatty acid desaturase
	hpnM	AFERRI_v2_10247	Hopanoid biosynthesis associated membrane protein
	hpnJ	AFERRI_v2_50952	Hopanoid biosynthesis associated radical SAM protein
	hpnK	AFERRI_v2_ 50953	Hopanoid biosynthesis
	hpnN	AFERRI_v2_50954	Hopanoid biosynthesis associated RND transporter
	hpnL	AFERRI_v2_50955	Membrane protein

	hpnH	AFERRI_v2_50956	Hopanoid biosynthesis associated radical SAM protein
	hpnM	AFERRI_v2_50957	Hopanoid biosynthesis associated membrane protein
Copper resistance	*copA*	AFERRI_v2_20792	Copper-exporting P-type ATPase A
	copZ	AFERRI_v2_20794	Putative cytoplasmic copper chaperone copZ
	copB	AFERRI_v2_40056	Copper-exporting P-type ATPase B
	cusA	AFERRI_v2_11213, AFERRI_v2_40050, AFERRI_v1_p0005	Cation efflux system protein, membrane component
	cusB	AFERRI_v2_11212, AFERRI_v2_40051, AFERRI_v1_p0004	Efflux transporter, RND family, MFP subunit
	cusC	AFERRI_v2_11211, AFERRI_v2_40052, AFERRI_v1_p0003	Outer membrane efflux protein
	cusF	AFERRI_v2_11214, AFERRI_v2_40048, AFERRI_v1_p0008	Periplasmic copper-binding protein (chaperone)
	ppx	AFERRI_v2_50972	Exopolyphosphatase
	pho84	AFERRI_v2_11093, AFERRI_v2_30142	Putative phosphate transporter (Pho84)
Zinc and manganese resistance	*corA*	AFERRI_v2_20852, AFERRI_v2_30191, AFERRI_v2_30192, AFERRI_v2_30193, AFERRI_v2_30194	$Mg^{2+}/Co^{2+}/Zn^{2+}$ transporter protein CorA family protein
	czcD	AFERRI_v2_10254, AFERRI_v2_40062, AFERRI_v2_40063, AFERRI_v2_50497	$Cd^{2+}/Co^{2+}/Zn^{2+}$ efflux protein
Mercury resistance	*merA*	AFERRI_v2_20196	Mercuric reductase
	merP	AFERRI_v2_20197	Periplasmic mercuric ion binding protein
	merT	AFERRI_v2_20198	Mercuric transport protein
	merR	AFERRI_v2_20195	Regulatory protein
Arsenic resistance	*arsC*	AFERRI_v2_10048, AFERRI_v2_30052	Arsenate reductase
	arsD	AFERRI_v2_10049	Trans-acting repressor and As^{3+}/Sb^{3+} chaperone

	arsA	AFERRI_v2_10050	Arsenical pump-driving ATPase
	arsB	AFERRI_v2_20030	As^{3+}/Sb^{3+} pump membrane protein
	arsR	AFERRI_v2_10047	Regulatory protein
Motility	*motB*	AFERRI_v2_50681	Flagellar motor rotation protein
	motA	AFERRI_v2_50682	Flagellar motor rotation protein
	fliL	AFERRI_v2_50683	Flagellar basal body-associated protein
	flhA	AFERRI_v2_50684	Flagellar biosynthesis protein
	flhB	AFERRI_v2_50685	Flagellar biosynthesis protein
	fliR	AFERRI_v2_50686	Flagellar biosynthesis protein
	fliQ	AFERRI_v2_50687	Flagellar biosynthesis protein
	fliI	AFERRI_v2_50689	Flagellum-specific ATP synthase
	fliH	AFERRI_v2_50690	Flagellar assembly protein
	flgA	AFERRI_v2_50691	Flagellar basal-body P-ring formation protein
	fliA	AFERRI_v2_50694	RNA polymerase sigma factor 28 for flagellar operon
	fliQ	AFERRI_v2_50695	Flagellar biosynthesis protein
	fliP	AFERRI_v2_50696	Flagellar biosynthesis protein
	fliN	AFERRI_v2_50697	Flagellar motor switch protein
	fliM	AFERRI_v2_50698	Flagellar motor switch protein
	fliG	AFERRI_v2_50700	Flagellar motor switch protein
	fliF	AFERRI_v2_50701	Flagellar M-ring protein

	fliE	AFERRI_v2_50702	Flagellar basal-body component
	fliS	AFERRI_v2_50703	Flagellin-specific chaperone
	fliD	AFERRI_v2_50704	Flagellar hook-associated protein
	fliC	AFERRI_v2_50705	Flagellin protein
	flgL	AFERRI_v2_50709	Flagellar hook-associated protein
	flgK	AFERRI_v2_50710	Flagellar hook-associated protein
	flgJ	AFERRI_v2_50711	Peptidoglycan hydrolase
	flgI	AFERRI_v2_50712	Flagellar basal body P-ring
	flgH	AFERRI_v2_50713	Flagellar L-ring protein
	flgG	AFERRI_v2_50714	Flagellar component of cell-distal portion of basal-body rod
	flgF	AFERRI_v2_50715	Flagellar basal-body rod protein
	flgE	AFERRI_v2_50716	Flagellar hook protein
	flgD	AFERRI_v2_50718	Flagellar hook capping protein
	flgC	AFERRI_v2_50719	Flagellar component of cell-proximal portion of basal-body rod
	flgB	AFERRI_v2_50720	Flagellar basal body rod protein
Chemotaxis	*mcp1*	AFERRI_v2_50673	Methyl-accepting chemotaxis protein
	cheV	AFERRI_v2_50674	Chemotaxis protein
	mcp2	AFERRI_v2_50675	Methyl-accepting chemotaxis protein
	mcp3	AFERRI_v2_50677	Methyl-accepting chemotaxis protein
	cheA	AFERRI_v2_50678	Signal transduction histidine kinase CheA

	cheZ	AFERRI_v2_50679	Chemotaxis phosphatase
	cheY	AFERRI_v2_50680	Chemotaxis regulator transmitting signal to flagellar motor component

Supplementary Table S6. Specific gene clusters of *At. ferrivorans* CF27 and their characteristics. CAI, codon adaptation index; PI, protein isolectric point. The topology is given as the position of the transmembrane helices separated by "i" if the loop is on the inside or "o" if it is on the outside. As example, i23-43o56-75i means that it starts on the inside, has a predicted TMhelix at position 21 to 43, the outside, then a TMhelix at position 56 to 75, ….

**Cluster number**	**Number of genes**	**GC contents (%)**	**Gene orientation**	**Gene ID**	**Length (bp)**	**Cellular localization from MicroScope**	**Membrane topology of the protein**	**CAI**	**PI**
1	5	54	---++	AFERRI_v2_10201	171	Unknown		0.6	5.6
				AFERRI_v2_10202	192	Unknown		0.7	10.6
				AFERRI_v2_10203	222	Unknown		0.6	12.0
				AFERRI_v2_10204	141	Unknown		0.7	9.4
				AFERRI_v2_10205	351	Cytoplasmic		0.7	4.7
2	4	46	++++	AFERRI_v2_10427	267	Unknown		0.7	9.1
				AFERRI_v2_10428	246	Unknown		0.6	4.1
				AFERRI_v2_10429	168	Unknown		0.6	10.6
				AFERRI_v2_10430	180	Transmembrane protein	i12-34o	0.7	5.3
3	6	42	+-+++-	AFERRI_v2_10437	231	Transmembrane protein	i21-43o56-75i	0.5	11.0
				AFERRI_v2_10438	183	Unknown		0.6	9.7
				AFERRI_v2_10439	1293	Unknown		0.6	8.3
				AFERRI_v2_10440	213	Transmembrane protein	o5-27i34-68o	0.4	9.8
				AFERRI_v2_10441	321	Transmembrane protein	i2-24o29-51i56-78o83-105i	0.4	10.8
				AFERRI_v2_10442	147	Cytoplasmic		0.5	3.5
4	3	50	+--	AFERRI_v2_10448	477	Transmembrane protein	i33-55o	0.7	8.9

				AFERRI_v2_10449	417	Cytoplasmic		0.6	9.8
				AFERRI_v2_10450	132	Unknown		0.4	11.1
5	3	51	---	AFERRI_v2_10521	384	Transmembrane protein	i45-67o77-99i	0.7	6.1
				AFERRI_v2_10522	237	Unknown		0.6	11.0
				AFERRI_v2_10523	381	Transmembrane protein	i39-58o80-102i	0.6	10.4
6	6	52	+---++	AFERRI_v2_10588	333	Unknown		0.6	9.5
				AFERRI_v2_10589	195	Cytoplasmic		0.7	4.7
				AFERRI_v2_10590	177	Cytoplasmic		0.5	11.8
				AFERRI_v2_10591	213	Unknown		0.6	4.8
				AFERRI_v2_10592	264	Unknown		0.5	11.5
				AFERRI_v2_10593	417	Unknown		0.7	9.4
7	4	51	----	AFERRI_v2_10615	348	Unknown		0.7	7.1
				AFERRI_v2_10616	222	Unknown		0.6	4.9
				AFERRI_v2_10617	342	Unknown		0.5	9.0
				AFERRI_v2_10618	210	Unknown		0.6	9.5
8	4	51	---+	AFERRI_v2_10631	492	Cytoplasmic		0.7	8.5
				AFERRI_v2_10632	576	Unknown		0.6	9.3
				AFERRI_v2_10633	666	Cytoplasmic		0.6	9.1
				AFERRI_v2_10634	720	Transmembrane protein	i97-119o139-161i	0.7	9.3
9	4	48	+-++	AFERRI_v2_10737	231	Unknown		0.5	5.8
				AFERRI_v2_10738	309	Unknown		0.6	5.0

				AFERRI_v2_10739	246	Unknown		0.6	10.0
				AFERRI_v2_10740	138	Unknown		0.6	9.6
10	3	55	++-	AFERRI_v2_10835	771	Transmembrane protein	o10-32i78-97o	0.6	8.7
				AFERRI_v2_10836	705	Transmembrane protein	o20-42i85-107o117-139i	0.6	7.4
				AFERRI_v2_10837	339	Transmembrane protein	o15-36i57-79o	0.7	7.0
11	3	45	+++	AFERRI_v2_11035	138	Unknown		0.6	6.9
				AFERRI_v2_11036	255	Unknown		0.6	5.3
				AFERRI_v2_11037	135	Transmembrane protein	i13-30o	0.7	10.0
12	3	46	++-	AFERRI_v2_20087	291	Unknown		0.7	4.5
				AFERRI_v2_20088	201	Unknown		0.6	4.5
				AFERRI_v2_20089	129	Unknown		0.6	6.0
13	3	56	+++	AFERRI_v2_20278	309	Cytoplasmic		0.6	4.9
				AFERRI_v2_20279	99	Unknown		0.5	5.5
				AFERRI_v2_20280	228	Cytoplasmic		0.7	5.0
14	3	60	---	AFERRI_v2_20301	192	Unknown		0.6	8.8
				AFERRI_v2_20302	270	Cytoplasmic		0.6	5.3
				AFERRI_v2_20303	297	Cytoplasmic		0.7	5.0
15	3	54	+--	AFERRI_v2_20984	546	Unknown		0.6	11.3
				AFERRI_v2_20985	627	Cytoplasmic		0.7	5.5
				AFERRI_v2_20986	114	Unknown		0.6	6.5
16	3	57	--+	AFERRI_v2_21033	63	Unknown		0.7	6.8

				AFERRI_v2_21034	255	Unknown		0.6	11.2
				AFERRI_v2_21035	477	Unknown		0.6	10.2
17	3	49	+++	AFERRI_v2_30310	138	Transmembrane protein	o4-26i	0.5	11.0
				AFERRI_v2_30311	135	Transmembrane protein	i7-29o	0.6	6.8
				AFERRI_v2_30312	906	Transmembrane protein	i12-34o49-71i121-143o153-170i	0.6	9.5
18	3	37	++-	AFERRI_v2_40073	183	Cytoplasmic		0.5	8.3
				AFERRI_v2_40074	570	Transmembrane protein	i39-58o62-84i97-116o155-177i	0.5	9.1
				AFERRI_v2_40075	678	Unknown		0.5	9.3
19	3	48	-++	AFERRI_v2_50812	228	Cytoplasmic		0.6	10.2
				AFERRI_v2_50813	222	Unknown		0.7	7.1
				AFERRI_v2_50814	624	Unknown		0.6	9.7

Supplemantary Table S7. Genes predicted to encode phage, transposon or plasmid proteins detected in the vicinity of CF27 specific gene clusters. White cells: absence of the gene encoding the indicated protein; black cells: presence of the gene encoding the indicated protein; the AFERRI_v2_ number is indicated in white letterings.

Protein/ Cluster	Phage assembly protein	Reverse transcriptase	Phage type recombinase	Phage type endonuclease	HNH endonuclease	Relaxase	Integrase	Resolvase	Transposase	Conjugation protein	Mobilization protein	Plasmid protein
1						10208					10199	
2												
3	10444											
4	10444											
5									10528			
6									10581	10587		
7										10602		
8					10638							
9					10750							10729
10										10832-10834		
11										11028		
12			20084	20082			20080					
13							20272					
14								20304	20308			
15									21006, 21009			
16		21039					21023					21030
17		30302							30296, 30319			
18							40072	40086				40079
19							50821					

Supplementary Table S8. RT-PCR on some CF27 specific genes. In the second column, is illustrated the cluster organization as presented in MicroScope (Vallenet et al., 2017). In red, the genes which expression has been analyzed. The RT-PCR experiments were given in the third column. RT-PCR experiments with oligonucleotides listed in Table S1 on total RNA from CF27 Fe(II)-grown cells (F) or sulfur attached cells (S) without reverse transcriptase (-), with reverse transcriptase (+), on CF27 genomic DNA (D). M is the 1 kb plus DNA ladder from invitrogen. Below the photo are indicated the concentration of the RNA used to perform the RT-PCR experiment and the size of the expected RT-PCR product in parenthesis. The green dot at the right of a band indicated that this band has been PCR amplified and sequenced. The conclusions are indicated in the right column.

Clusters	Organisation and RT-PCR	Results	Conclusions
rrs		F S<br>M - + + - M<br>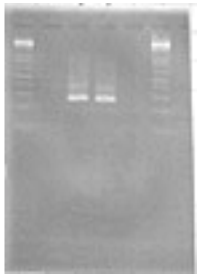<br>0.1 ng RNA (277 bp)	*rrs* gene is transcribed to the same extent in Fe(II)-grown and sulfur attached cells
1	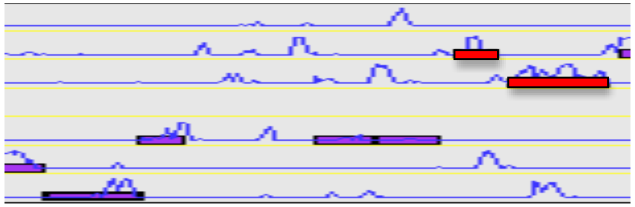	F S<br>M - + - +<br>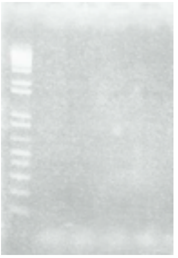<br>15 ng RNA (446 bp)	10204 and 10205 are not cotranscribed in Fe(II)-grown and sulfur attached cells under the conditions utilised

	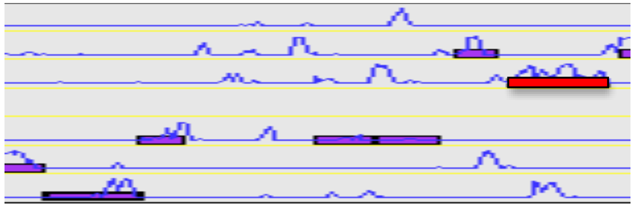	F S F S<br>M - - D + + M<br>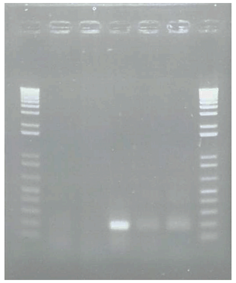<br>10ng RNA (187 bp)	10205 is slightly more transcribed in sulfur attached than in Fe(II)-grown cells
2	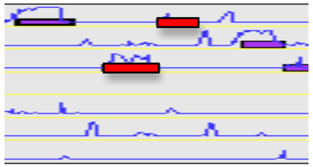<br>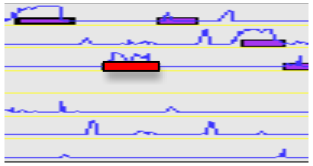	F<br>- + M<br>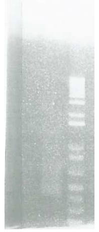<br>10 ng RNA (247 bp)<br>F S F S<br>M - - D + + M<br>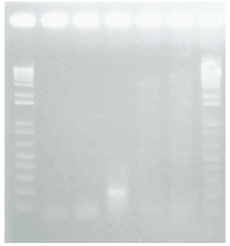<br>50 ng RNA (205 bp)	10428 and 10429 are not cotranscribed in Fe(II)-grown cells under the conditions utilised<br><br>10428 is not transcribed in Fe(II)-grown and sulfur attached cells under the conditions utilised

3	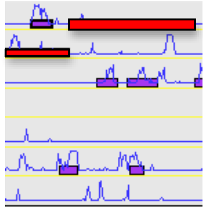<br>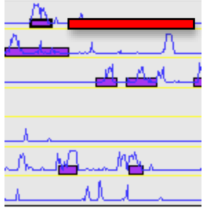	F<br>- + M<br>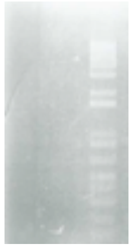<br>20 ng RNA (403 bp)<br>F S F S<br>M - - D + + M<br>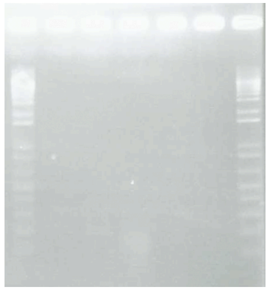<br>50 ng RNA (180 bp)	10436 and 10439 are not cotranscribed in Fe(II)-grown cells under the conditions utilised<br>10439 is not transcribed in Fe(II)-grown and sulfur attached cells under the conditions utilised
4	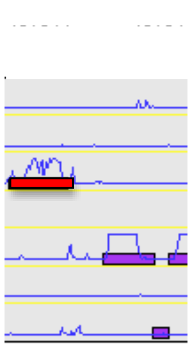	F S<br>M - + + - D M<br><br>10 ng RNA (220 bp)	10448 is transcribed in Fe(II)-grown and sulfur attached cells to the same extent

5	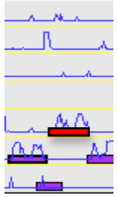	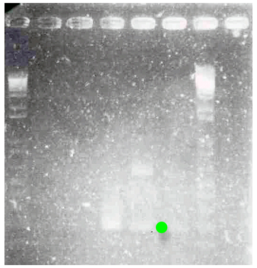<br>10 ng RNA (179 bp)	10523 is transcribed in Fe(II)-grown and sulfur attached cells to the same extent
6	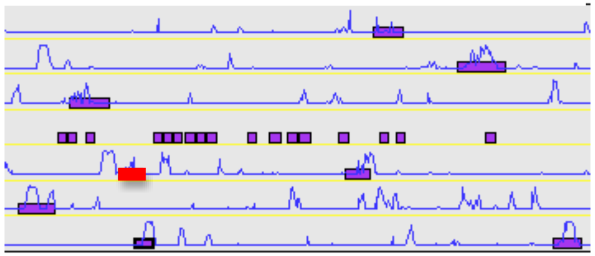	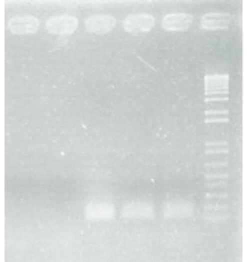<br>5 ng RNA (167bp)	10589 is transcribed in Fe(II)-grown and sulfur attached cells to the same extent
7	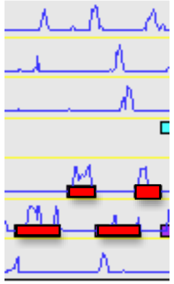	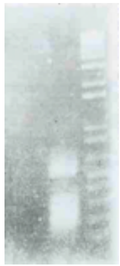<br>5 ng RNA (825 bp)	10615, 10616, 10617 and 10618 are not cotranscribed in Fe(II)-grown cells under the conditions utilised

	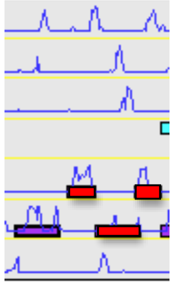	F S F S<br>M - - D + + M<br>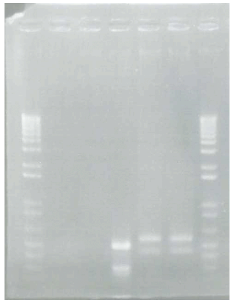<br>5 ng RNA (568 bp)	10616, 10617 and 10618 are not cotranscribed in Fe(II)-grown and sulfur attached cells under the conditions utilised
	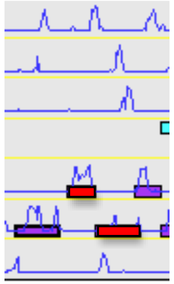	F S<br>- + + - M<br>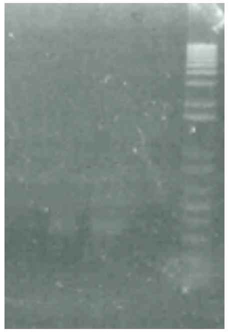<br>1 ng RNA (315 bp)	10616 and 10617 are cotranscribed in Fe(II)-grown and sulfur attached cells to the same extent

8	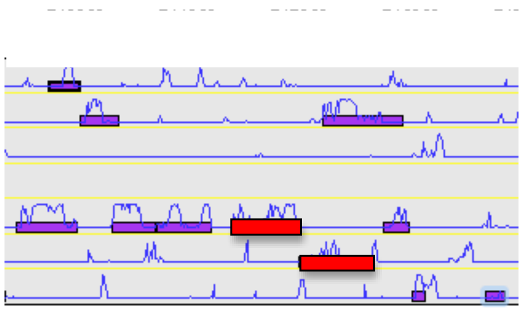<br>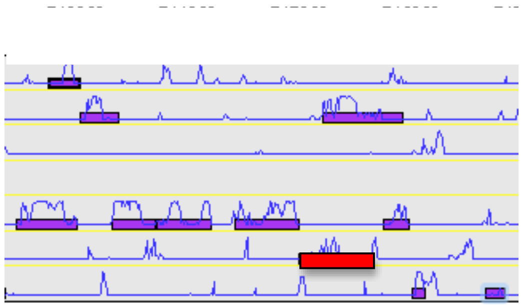<br>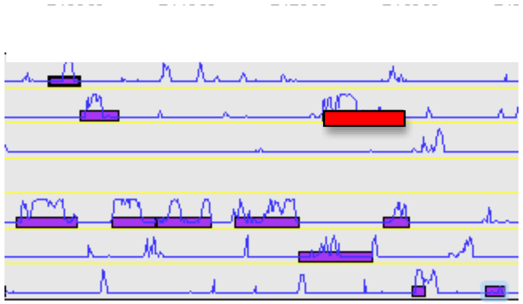	F<br>- + M<br>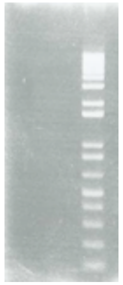<br>9 ng RNA (293 bp)<br>F<br>- + M<br>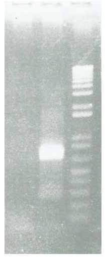<br>1 ng RNA (164 bp)<br>F S<br>- + + - M<br>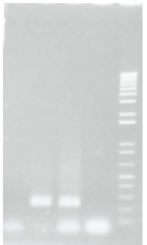<br>5 ng RNA (246 bp)	10632 and 10633 are not cotranscribed in Fe(II)-grown cells under the conditions utilised<br>10633 is not transcribed in Fe(II)-grown cells under the conditions utilised<br>10634 is transcribed in Fe(II)-grown and sulfur attached cells to the same extent

9	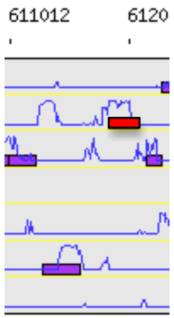	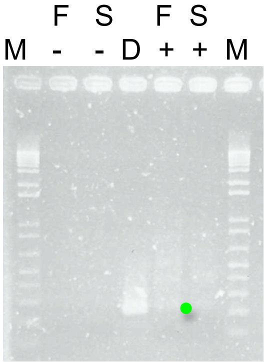<br>5 ng RNA (227 bp)	10739 is transcribed in Fe(II)-grown and sulfur attached cells to the same extent
10	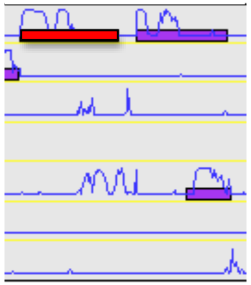	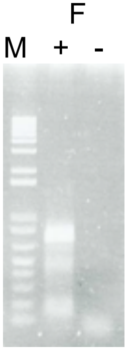<br>10 ng RNA (355 bp)	10835 is not transcribed in Fe(II)-grown cells under the conditions utilised
11	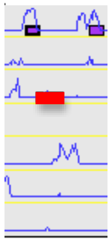	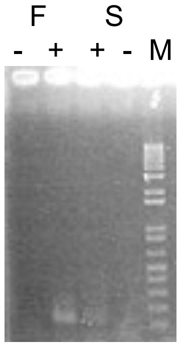<br>5ng RNA (134 bp)	11036 is more transcribed in Fe(II)-grown than in sulfur attached cells

12	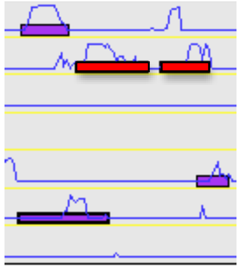<br>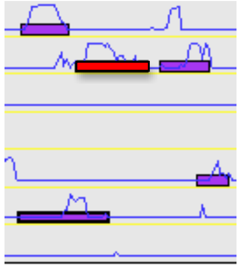	F<br>M - +<br>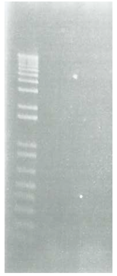<br>5 ng RNA (361 bp)<br>F<br>M + -<br>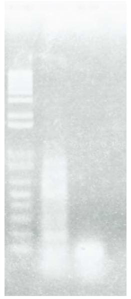<br>10 ng RNA (227 bp)	20087 and 20088 are not cotranscribed in Fe(II)-grown cells under the conditions utilised<br>20087 is not cotranscribed in Fe(II)-grown cells under the conditions utilised

	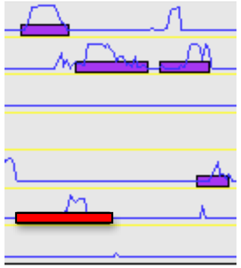	F S F S<br>M - - D + + M<br>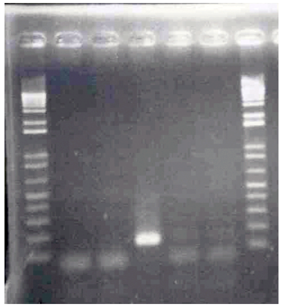<br>5 ng RNA(149 bp)	340015 is transcribed in Fe(II)-grown and sulfur attached cells to the same extent
13	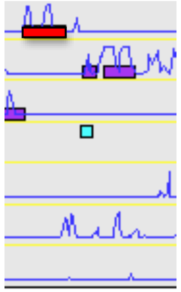	F S F S<br>M + + D - - M<br>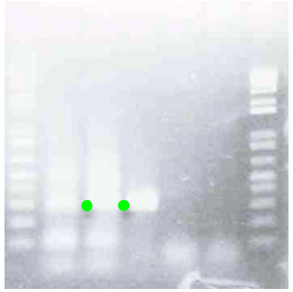<br>50 ng (302 bp)	20278 is transcribed in Fe(II)-grown and sulfur attached cells to the same extent

14	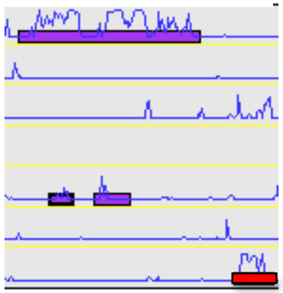	F S F S<br>M - - D + + M<br>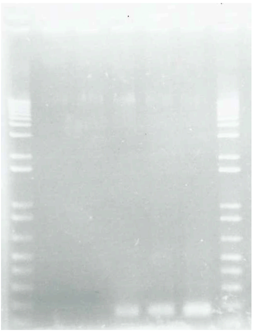<br>1ng (170 bp)	20303 is slightly more transcribed in sulfur attached than in Fe(II)-grown cells
15	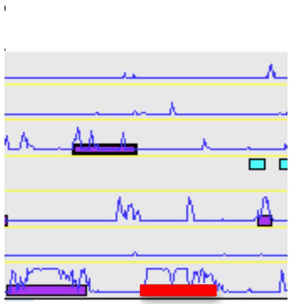	F S<br>M - + + - M<br>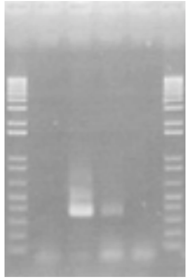<br>5 ng RNA (368 bp)	20985 is more transcribed in Fe(II)-grown than in sulfur attached cells
16	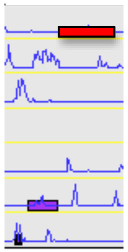	F S<br>M - + + -<br>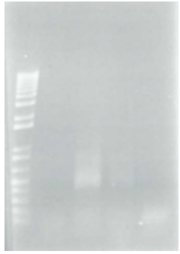<br>10 ng RNA (421 bp)	21035 is more transcribed in Fe(II)-grown than in sulfur attached cells

17	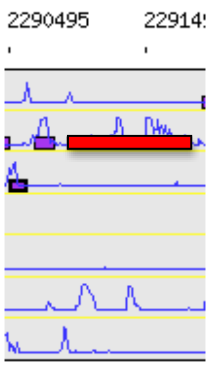	F S F S<br>D + + - - M<br>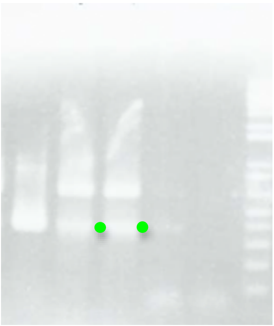<br>10ng RNA (372 bp)	30312 is transcribed in Fe(II)-grown and sulfur attached cells to the same extent
18	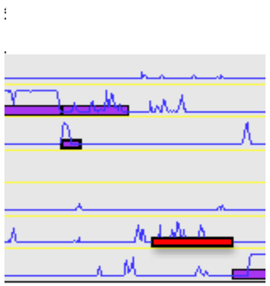	F S F S<br>M + + D - - M<br><br>50 ng (306 bp)	40075 is not transcribed in Fe(II)-grown and sulfur attached cells under the conditions utilised
19	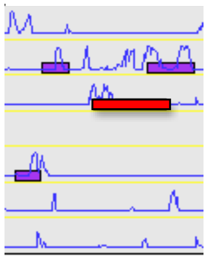	F S<br>M - + + - M<br>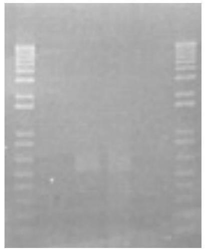<br>5 ng RNA (562 bp)	50814 is transcribed in Fe(II)-grown and sulfur attached cells to the same extent

Vallenet, D., Calteau, A., Cruveiller, S., Gachet, M., Lajus, A., Josso, A., Mercier, J., Renaux, A., Rollin, J., Rouy, Z., Roche, D., Scarpelli, C., and Medigue, C. (2017). MicroScope in 2017: an expanding and evolving integrated resource for community expertise of microbial genomes. *Nucleic Acids Res*. 45 (D1): D517-D528. doi: 10.1093/nar/gkw1101.

Supplementary Table S9. The taxomonic affiliation of top-scoring BLAST hits of proteobacteria phyla against the complete prokaryotic dataset proteins. For each phyla, the numbers indicate the average of percentage of protein ± standard deviations.

**Best BLASTP assignment**		***Acidithiobacillus* spp.**	**Alphaproteobacteria**	**Betaproteobacteria**	**Deltaproteobacteria**	**Gammaproteobacteria**
Bacteria	Gammaproteobacteria	**31.0±2.2**	2.3±2.0	5.9±4.1	4.1±3.2	**89.8±5.4**
	Betaproteobacteria	**27.3±2.1**	1.9±1.6	**81.5±9.1**	1.9±1.0	1.7±1.8
	Alphaproteobacteria	5.7±0.8	**86.1±7.7**	2.0±1.7	1.4±0.6	1.0±1.3
	Deltaproteobacteria	2.7±0.3	0.4±0.4	1.1±0.9	**70.2±15.2**	0.4±0.6
	Epsilonproteobacteria	0.3±0.1	0	0.1±0.1	0.4±0.4	0.2±0.5
	Firmicutes	2.2±0.4	0.2±0.2	0.3±0.2	3.5±2.9	0.5±0.4
	Cyanobacteria	1.8±0.3	0.3±0.3	0.5±0.5	1.3±0.8	0.2±0.3
	Actinobacteria	0.7±0.2	0.6±0.6	0.3±0.3	0.5±0.5	0.2±0.2
	Chlamydiae	0.1±0.1	0	0	0.1±0.1	0
	Bacteroidetes	0.5±0.1	0.3±0.3	0.2±0.2	0.9±0.5	0.2±0.3
	Spirochaetes	0.1±0.1	0	0	0.4±0.3	0.1±0.1
	Deinococcus-Thermus	0.3±0.1	0.1±0.1	0.1±0.1	0.2±0.1	0
	Thermotogae	0.1±0.1	0	0	0.1±0.1	0
	Chloroflexi	0.4±0.1	0.1±0.1	0.1±0.1	0.5±0.5	0
	Aquificae	0.7±0.1	0	0	0.2±0.3	0
	Chlorobi	0.5±0.2	0	0.1±0.1	0.6±0.4	0.1±0.2
	Fusobacteria	0	0	0	0.1±0.1	0.1±0.1
	Acidobacteria	0.6±0.2	0.2±0.2	0.1±0.1	0.3±0.3	0
	Planctomycetes	0.2±0.1	0.1±0.1	0.1±0.1	0.3±0.2	0
	Synergistetes	0	0	0	0.2±0.2	0

	Verrucomicrobia	0.2±0.1	0.0±0.1	0.0±0.1	0.1±0.1	0
	Nitrospirae	1.2±0.2	0	0.1±0.2	0.5±0.6	0
	Deferribacteres	0.1±0.1	0	0	0.4±0.3	0
	Thermodesulfobacteria	0.1±0.1	0	0	0.8±1.0	0
	Ignavibacteriae	0	0	0	0.2±0.1	0
	Chrysiogenetes	0.1±0.0	0	0	0.2±0.5	0
	CandidatedivisionNC10	0.1±0.1	0	0.0±0.1	0.2±0.3	0
Archaea	Euryarchaeota	0.5±0.1	0.1±0.1	0.1±0.1	1.1±0.9	0.0±0.1
	Crenarchaeota	0.1±0.1	0	0	0.1±0.1	0
NO BLAST		**22.4±4.8**	7.2±3.6	7.2±3.5	9.1±4.2	5.3±3.4
