# Supplementary material for: Comparative Genome Analysis Provides Insights into Both the Lifestyle of Acidithiobacillus ferrivorans Strain CF27 and the Chimeric Nature of the Iron-Oxidizing Acidithiobacilli Genomes
Source: Front Microbiol. 2017 Jun 13;8:1009. doi: 10.3389/fmicb.2017.01009 (PMC5468388; doi:10.3389/fmicb.2017.01009)
Supplement: Supplementary file 3 [file Data_Sheet_3.DOCX]

**Supplementary Text**

**Script 1.** ***Calculate_gc_content_for_circos.pl***

#!/usr/bin/perl

# usage: *Calculate_gc_content_for_circos.pl file.fasta*

# file = chromosome or plasmid in fasta format

if (@ARGV !=1) {

print "Usage: $0 fasta_file!\n";

} else {

$file = $ARGV[0];

}

$file = @ARGV[0];

$fasta_sequence = qx(more $file | grep -v ">" | tr -d '\n' ); *# Remove the description line*

$length = length($fasta_sequence);

$pos1 =1;

while($pos1 < $length) {

$pos2= $pos1 + 4999;

system("extractseq -stdout -sequence $file -regions [$pos1-$pos2] -auto > seqout$pos1"); # extractseq from Emboss package

my $gc= qx(infoseq -sequence seqout$pos1 -only -pgc -auto | tail -n 1); chomp($gc); # infoseq from Emboss package

my $location = $pos1 -1;

print"AFERRI_v2\t$pos1\t$pos2\t$gc\n";#AFERRI_v2 should be changed to the name of subject

system("rm seqout$pos1");

$pos1 = $pos1 +1000;

}

**Script 2.** ***Calculate_gc_skew_for_circos.pl***

#!/usr/bin/perl

# usage: *Calculate_gc_skew_for_circos.pl file.fasta*

# file = chromosome or plasmid in fasta format

if (@ARGV !=1) {

print "Usage: $0 fasta_file!\n";

} else {

$file = $ARGV[0];

}

$file = @ARGV[0];

$fasta_sequence = qx(more $file | grep -v ">" | tr -d '\n' ); # Remove the description line

$length = length($fasta_sequence);

$pos1 =1;

while($pos1 < $length) {

$pos2= $pos1 + 4999;

my $sequence = qx(extractseq -stdout -sequence $file -regions [$pos1-$pos2] -auto| grep -v ">");

my $c = ($sequence =~ tr/C/c/); # count up Cs

my $g = ($sequence =~ tr/G/g/); # count up Gs;

my $skew = ($c-$g)/($c+$g)*100; # Calculate GC skew

my $GCSkew = sprintf( "%.2f", $skew );

print"AFERRI_v2\t$pos1\t$pos2\t$GCSkew\n"; #AFERRI_v2 should be changed to the name of subject

$pos1 = $pos1 +1000;

}
